# Strong coupling in plasmonic metal nanoparticles

**DOI:** 10.1186/s40580-023-00383-5

**Published:** 2023-07-20

**Authors:** Yoon-Min Lee, Seong-Eun Kim, Jeong-Eun Park

**Affiliations:** Department of Chemistry, Gwangju Institute of Science and Technology, Gwangju, 61005 Korea

**Keywords:** Light-matter interaction, Strong coupling, Polaritons, Metal nanoparticle, Plasmonics

## Abstract

The study of strong coupling between light and matter has gained significant attention in recent years due to its potential applications in diverse fields, including artificial light harvesting, ultraefficient polariton lasing, and quantum information processing. Plasmonic cavities are a compelling alternative of conventional photonic resonators, enabling ultracompact polaritonic systems to operate at room temperature. This review focuses on colloidal metal nanoparticles, highlighting their advantages as plasmonic cavities in terms of their facile synthesis, tunable plasmonic properties, and easy integration with excitonic materials. We explore recent examples of strong coupling in single nanoparticles, dimers, nanoparticle-on-a-mirror configurations, and other types of nanoparticle-based resonators. These systems are coupled with an array of excitonic materials, including atomic emitters, semiconductor quantum dots, two-dimensional materials, and perovskites. In the concluding section, we offer perspectives on the future of strong coupling research in nanoparticle systems, emphasizing the challenges and potentials that lie ahead. By offering a thorough understanding of the current state of research in this field, we aim to inspire further investigations and advances in the study of strongly coupled nanoparticle systems, ultimately unlocking new avenues in nanophotonic applications.

## Introduction

The interaction between light and matter is fundamental to a wide range of fields, from basic sciences to advanced technologies. When molecules or materials are exposed to light, they interact with it, changing the light energy mainly through absorption, scattering, reflection, or emission. This interaction enables us to explore, improve or utilize their properties. Recently, strong coupling between light and matter has gained attention due to its various potential applications, such as artificial light harvesting, ultraefficient lasing, and quantum information processing. In contrast to conventional weak coupling between light and matter, strong coupling occurs when coupling strength (g) overcomes the losses of each component. This results in the formation of new states called polaritons. For instance, when an emitter is placed in an optical resonator that supports a sufficient electromagnetic field, the hybridization between photons and excitons can create exciton-polaritons, possessing both excitonic and photonic properties [1–4]. This leads to a mode splitting, as in molecular orbital theory, and the Rabi splitting (Ω) corresponds to the spectral separation of normal modes when the photon and the exciton are in perfect resonance. The strength of the coupling can be quantified through this Rabi splitting in scattering or photoluminescence (PL) spectra [5]. In addition to the Rabi splitting observed in optical spectra, an interesting phenomenon known as the anti-crossing of polaritonic dispersion curves can also be observed in the energy–momentum diagram. At the point of intersection, the plasmon and exciton modes exhibit a repulsive behavior, indicating a strong interaction between them. However, it's worth noting that this review primarily focuses on Rabi splitting, as spectrum measurements, not dispersion diagrams, serve as the main characterization method for nanoparticle systems. Purcell effect, which speeds up emission and increases quantum efficiency in a weak coupling regime, has also been observed even in strongly coupled systems [6].

Traditional photonic resonators like Fabry-Pérot (FP) cavities [7–10] and photonic crystals [11–15] offer low damping loss. However, they are incapable of compressing mode volumes below the diffraction limit. This limitation hinders the enhancement of coupling strength, as the coupling strength, $\mathrm{g}\propto \frac{1}{\sqrt{\mathrm{V}}}$ where V is the mode volume. While operating at cryogenic temperatures can help overcome this issue, it introduces technical challenges and limits the practical implementation, scalability, and complexity of devices. As an alternative, plasmonic cavities offer nanoscale mode volumes, despite their low-quality factor, making them an appealing platform for ultracompact polaritonic systems that can function at room temperature.

Among the variety types of plasmonic cavities, our focus is on colloidal metal nanoparticles, which support localized surface plasmon resonance (LSPR) due to the collective oscillations of electrons at their surfaces. They offer the advantage of easy synthesis in well-defined sizes and shapes, compared to lithographically fabricated plasmonic nanostructures. Additionally, their plasmonic properties can be easily modified by changing the size, geometry, and arrangement of the metallic nanoparticles. For instance, anisotropic nanoparticles, such as cubes and rods, can generate strong near-fields at corners or tips. The junction between multiple nanoparticles can produce gap plasmon modes with even greater near-field enhancement. Colloidal nanoparticles can display lower ohmic losses due to their higher crystallinity compared to top-down structures [16]. In the past two decades, metal nanoparticles have been shown to significantly influence the behavior of molecules and emitters in close proximity, as demonstrated by advancements in surface-enhanced Raman spectroscopy (SERS) and surface-enhanced fluorescence (SEF) [17, 18].

**Fig. 1:**
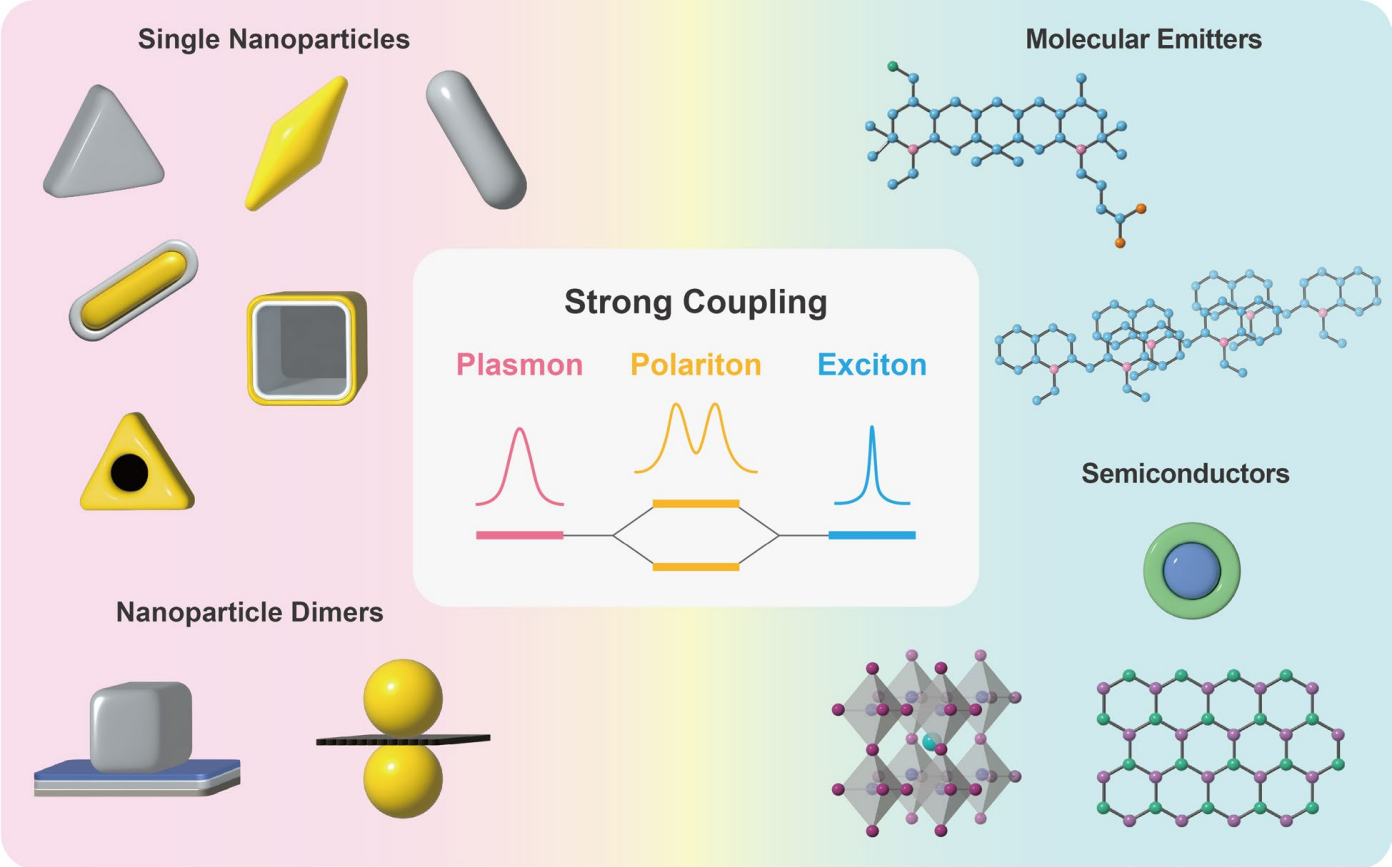
Formation of polaritons from strong coupling between plasmons and excitons (center) and representative plasmonic and excitonic materials

In this review, we discuss recent examples of strongly coupled systems operating at room temperature that incorporate emitters with plasmonic nanoparticle-based optical cavities (Fig. 1). Compared to low temperature, room temperature environments often introduce challenges such as higher levels of noise, scattering, and dissipation, which can impact the quality and stability of strong coupling interactions. Moreover, the temperature-dependent dissipation of plasmons possess additional challenges, as the damping of plasmons increases with rising temperatures, which can limit the performance and efficiency of coupled systems [19]. Despite these challenges, strong coupling at room temperature offers energy-efficient operation without requiring additional energy consumption or specialized cooling devices to maintain low temperatures, also offering practicality and compatibility with real-world applications. The experimental realizations covered in this paper include individual single nanoparticle colloids and dimeric nanoparticles, which are coupled with diverse excitonic materials such as atomic emitters, semiconductor quantum dots, and two-dimensional (2D) materials. Other types of plasmonic cavities, like 2D plasmonic nanoparticle lattices, are covered in separate review articles [20].

## Strong coupling in single nanoparticles

We first start with individual metal nanoparticles, which represent the simplest form of optical cavities. One of the most remarkable features of these nanoparticles is their flexibility in terms of optical properties, which can be easily tailored by adjusting parameters such as material composition, size, and shape. To facilitate a deeper understanding, we have divided the content into different categories based on the type of excitonic materials involved, each of which is coupled with a variety of Au, Ag, or bimetallic nanoparticles. By doing so, we aim to provide a comprehensive overview of the field and insights that arise from the different combinations of these materials.

### Coupling with J-aggregates

Cyanine dyes have garnered significant attention in various studies owing to their utility in spectral sensitization and their potential applications in organic optoelectronic materials [21, 22]. These dyes tend to form aggregates when their solubility decreases or when they adsorb onto the surfaces of particles or substrates. The resulting J-aggregates exhibit a narrow line shape that is red-shifted in comparison to the absorption band of the monomer, providing an advantage in achieving strong coupling. Additionally, organic molecular assemblies are considered soft compared to inorganic materials [23], making them ideal candidates for acting as excitonic shells surrounding plasmonic core nanoparticles. This configuration enables the coupling of excitonic and plasmonic properties, giving rise to the formation of hybrid states that exhibit unique optical and electronic characteristics.

#### Au nanoparticles

Au is a well-known plasmonic material with excellent performance in the visible and near-infrared (NIR) spectral ranges, and superior chemical stability under ambient conditions [24]. Among many types of Au nanoparticles (AuNPs), Au nanorods (AuNRs) are attractive due to their adjustable morphology and broader tunable LSPR range compared to Au nanospheres, a result of their anisotropic properties [25, 26].

In the first example, Kumar et al. demonstrated strong coupling in a hybrid system based on AuNRs, revealing the role of the plasmon decay channel and mode volume in strong coupling [27]. They synthesized three distinct AuNRs with varying diameters and mode volumes but identical LSPR peaks through oxidation etching. The resulting LSPR peaks were resonant with the J-band maximum (~ 2.1 eV) of 5,6-dichloro-2[3-[5,6-dichloro-1-ethyl-3-(3-sulfopropyl)-2(3*H*)-benzimidazolidene]-1-propenyl]-1-ethyl-3-(3-sulfopropyl) benzimidazolium hydroxide (TDBC). TDBC dye is commonly used as cyanine dye in polariton studies due to its narrow linewidth and large oscillator strength when forming J-aggregate in an aqueous solution [28].

Subsequently, they prepared hybrid nanostructures comprising TDBC J-aggregates and three different AuNRs. The J-aggregates were attached to the surfaces of the AuNRs via electrostatic attraction between negatively charged TDBC dyes and the positively charged CTAB-capped AuNRs. The extinction spectrum exhibited two spectral branches, the upper polariton and the lower polariton. Single particle dark-field (DF) spectroscopy measurements demonstrated that the TDBC J-aggregates were strongly coupled to the AuNRs, with Rabi splitting increasing from 135 to 200 meV as the LSPR linewidth decreased. The coupling strength is also affected by the mode volume which determines the confinement of the electric field within a cavity. By achieving a small mode volume, a robust electric field is generated, leading to a greater concentration of light. This enables plasmonic nanoparticles to interact more efficiently with light and facilitates strong coupling [29–31]. Interestingly, as the mode volume decreased, Rabi splitting decreased despite the formation of a stronger electric field at a small mode volume. A comparison of the Rabi splitting functions of LSPR linewidth and mode volume revealed that the mode volume exerted a more significant impact on strong coupling than the linewidth.

**Fig. 2:**
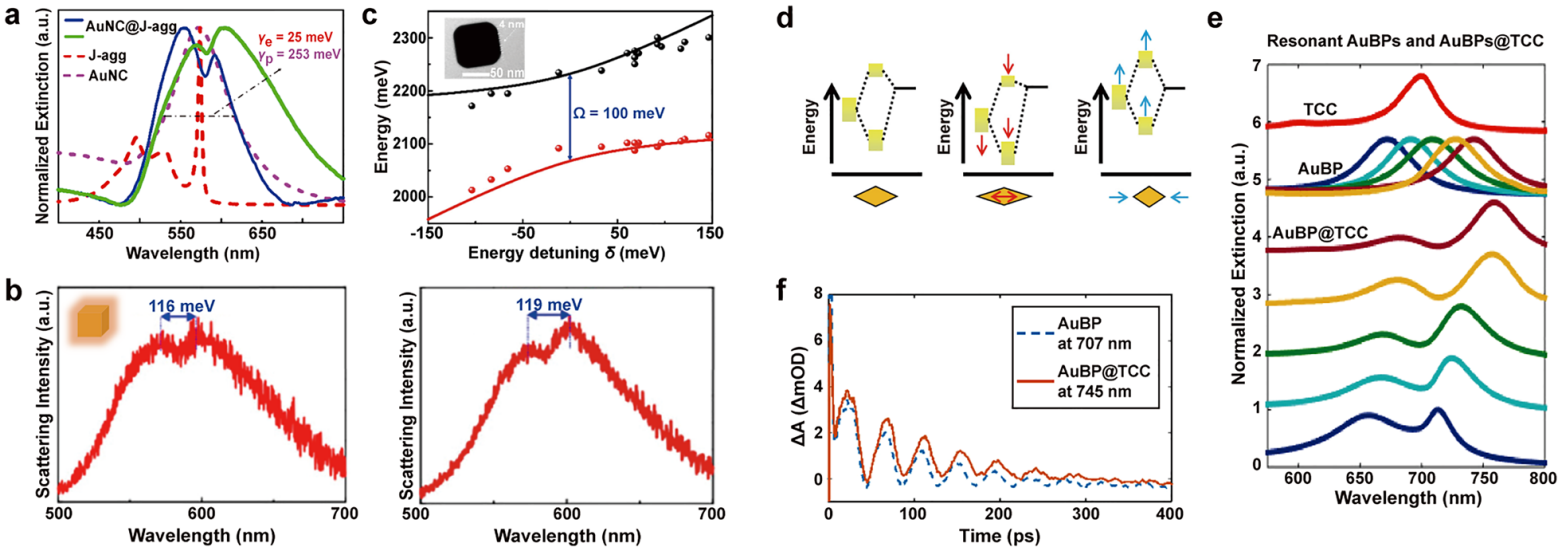
Strong coupling between Au nanoparticles and J-aggregates. **a** Extinction and **b** scattering spectra of AuNC@J-aggregates hybrids. **c** Anticrossing behavior curves of AuNC@J-aggregates hybrids. **d** The schematic energy diagrams of AuBPs with different aspect ratios. **e** Extinction spectra of AuBPs@TCC system with LSPRs that range from 640 to 750 nm. **f** Kinetic traces of the transient absorption spectrum for AuBP and the AuBP@TCC (95% confidence interval from fitting algorithm). Reproduced with permissions from [32, 37], respectively

A smaller mode volume can be translated into a large enhancement of the local field intensity, which can be observed around the sharp corners, edges, and conical tips of plasmonic nanoparticles [24]. Au nanocubes (AuNCs) present an increased electric field at edges and corners, and their flat surfaces make them beneficial building blocks [32]. Song et al. employed the J-aggregate-forming cyanine dye, 1,1′-diethyl-2,2′-cyanine iodide (PIC), to examine the strong coupling between AuNCs and J-aggregates. Similar to TDBC, PIC is also one of the extensively studied cyanine dyes that form J-aggregates. PIC exhibits a J-aggregates band at 570 nm, with a narrow spectral width (~ 33 meV) and large oscillator strength (f = 2.88) [33]. To attach the positively charged PIC J-aggregates to the AuNC surfaces, the CTAC-capped AuNCs with positive charge were first modified by introducing negative charge using Cl^−^ ions. Subsequently, the J-aggregates, formed in the presence of salt, were assembled on the Au surfaces through electrostatic attraction. The extinction and DF scattering spectra of AuNC@J-aggregates hybrids exhibited strong coupling, as evidenced by peak splitting with the Rabi splitting being 100 meV (Fig. 2a–c). Despite the sharp corners present in AuNCs compared to the ends of AuNRs, coupling with AuNRs led to a large Rabi splitting of 205 meV. This difference may occur because AuNRs exhibit a smaller linewidth (160 to 235 meV) in comparison to AuNCs (253 meV), but other conditions such as the linewidth of each J-aggregates and the energy mismatch between plasmons and excitons in each case need to be verified as well.

One can employ nanoparticles with void space surrounded by a J-aggregate layer for plasmon-exciton coupling. Hollow nanostructures can offer advantages for inducing strong coupling, as they provide increased surface area, which in turn leads to an increased interaction area between plasmons and excitons. Hollow Au nanoprisms (HGNs) have an anisotropic shape and a cavity at their center, making them unique nanostructures for plasmonic applications [34]. These nanoparticles not only increase the interaction area but also effectively localize and enhance the electric field of the incident light at their tips and center, thereby contributing to enhanced coupling between plasmons and excitons. By adjusting the aspect ratio of HGN, their LSPR was tuned to be resonant with J-aggregates, facilitating strong coupling between HGNs and PIC J-aggregates. The PIC dye molecules were adsorbed onto the HGN surfaces and self-assembled through π-π interactions. The decrease in zeta potentials of CTAB-capped HGN and HGN@PIC from 60.7 ± 5.5 mV to 46.9 ± 2.6 mV suggested that PIC dye molecule replaced the CTAB double layer. Extinction spectroscopy confirmed the occurrence of strong coupling in the HGN@J-aggregates hybrid system, exhibiting a Rabi splitting energy (Ω) of 198 meV and a coupling constant (g) of 91 meV. Local electric fields were simulated using the finite-difference time-domain (FDTD) method, revealing significant electric field enhancement within the HGN cavity. The simulated absorption, scattering, and extinction cross-sections of dye-coated HGNs showed strong agreement with experimental results. Compared to AuNCs, HGNs displayed greater Rabi splitting when employing the same dye, which could be attributed to the generation of a strong electric field not only at the tip but also within the central cavity of the HGN structure. Consequently, a structure that produces a strong electric field over an extensive area appears to be beneficial for achieving strong plasmon-exciton coupling.

Furthermore, the same group explored the roles of exciton oscillator strength and charge of J-aggregates by comparing HGN@J-aggregates hybrids with two cyanine dyes, TDBC and PIC [35]. TDBC and PIC dyes were selected due to their similar J-band peak positions at 2.1 eV and 2.17 eV, respectively, and narrow linewidths but with distinct oscillator strengths and charges. The adsorption of negatively charged TDBC dyes onto the surfaces of CTAB-capped HGNs primarily occurs through electrostatic attraction, whereas positively charged PIC dye molecules can be adsorbed onto HGNs through weak noncovalent interactions and self-assembled into J-aggregate structures because of the same charges of HGNs and PIC. The occurrence of strong coupling was observed in extinction and single-particle DF spectra of both HGN@PIC and HGN@TDBC composites with the Rabi splitting values for HGN@TDBC and HGN@PIC composites of 195 meV and 178 meV, respectively. When the surfaces of HGNs were further modified with a negatively charged polymer, polystyrene sodium sulfonate (PSS), the Rabi splitting value of the HGN@PIC hybrid increased from 178 to 201 meV. These results suggest that favorable electrostatic interactions enhance the number of excitons on the HGN surface, leading to large Rabi splitting value.

After confirming that strong coupling between HGNs and two J-aggregates, the effect of exciton oscillator strength on plasmon-exciton interaction was investigated. The aggregation numbers of the J-aggregates for each dye were determined to be 347 for PIC and 15 for TDBC J-aggregates (Aggregation number = [(FWHM of monomers/FWHM of J-aggregate)^2^]). Based on these numbers, the oscillator strength was calculated to be 2.94 and 0.51 for PIC and TDBC J-aggregates, respectively. The concentration-dependent Rabi splitting values for HGN@PIC and HGN@TDBC composites revealed that the Rabi splitting was higher for HGN@PIC than HGN@TDBC composites at dye concentrations up to 30 μM, possibly due to the larger oscillator strength of the PIC J-band at lower concentrations. However, at higher dye concentrations, the trend changed and HGN@TDBC exhibited higher Rabi splitting than HGN@PIC even with a larger oscillator strength of PIC than TDBC. The authors explained that owing to the planar structure of TDBC dye, a large number of dyes can adhere to the nanoparticle surface, resulting in significant Rabi splitting even though the oscillator strength is 5.7 times smaller than that of PIC.

Similar to previously discussed AuNRs, Au bipyramids (AuBPs) also exhibit two distinct surface plasmon resonances corresponding to the oscillations of electrons along the longitudinal and transverse directions. However, due to their sharper ends, AuBPs intensify local electric-field enhancements compared to nanorods [36]. Kirschner et al. employed a system comprising highly monodisperse AuBPs to create plasmon-exciton hybridized states [37]. AuBPs were synthesized with LSPRs ranging from 640 to 750 nm by adjusting their aspect ratio (Fig. 2d). The formation of J-aggregates of thiacarbocyanine dye, 2,2′-dimethyl-8-phenyl-5,6,5′,6′-dibenzothiacarbocyanine chloride (TCC) on the AuBP surfaces can be accomplished through van der Waals forces and electrostatic attraction. By functionalizing J-aggregates onto a series of AuBPs with varying aspect ratios and LSPR energies, two hybridized states were generated (Fig. 2e), displaying notable anticrossing behavior with a Rabi splitting energy of 120 meV. Then, the author analyzed the ultrafast dynamics of these systems to determine the effects of coherent acoustic phonons. In metal nanoparticles, coherent acoustic phonons are generated by photoexcitation, causing the LSPR to undergo an oscillatory redshift and blueshift [38, 39]. A similar phenomenon was observed in the case of AuBPs@TCC, where two sets of oscillating photoinduced absorptions and bleaches were observed, corresponding to the two polariton peaks. The oscillations were further illustrated by kinetic traces of the transient absorption spectrum for AuBP and the AuBP@TCC (Fig. 2f). The oscillation period of bare AuBP spectral oscillations at 707 nm was 42.52 ± 0.16 ps while that of the functionalized AuBPs at 745 nm was 43.61 ± 0.14 ps. The slight shift in the oscillation period observed in the AuBP@TCC system was attributed to the small additional mass of the dye adsorbed to the particles. This emphasizes the potential of AuBPs in mass sensing applications, where even a small amount of additional mass can lead to detectable changes in the oscillation period.

#### Ag nanoparticles

**Fig. 3:**
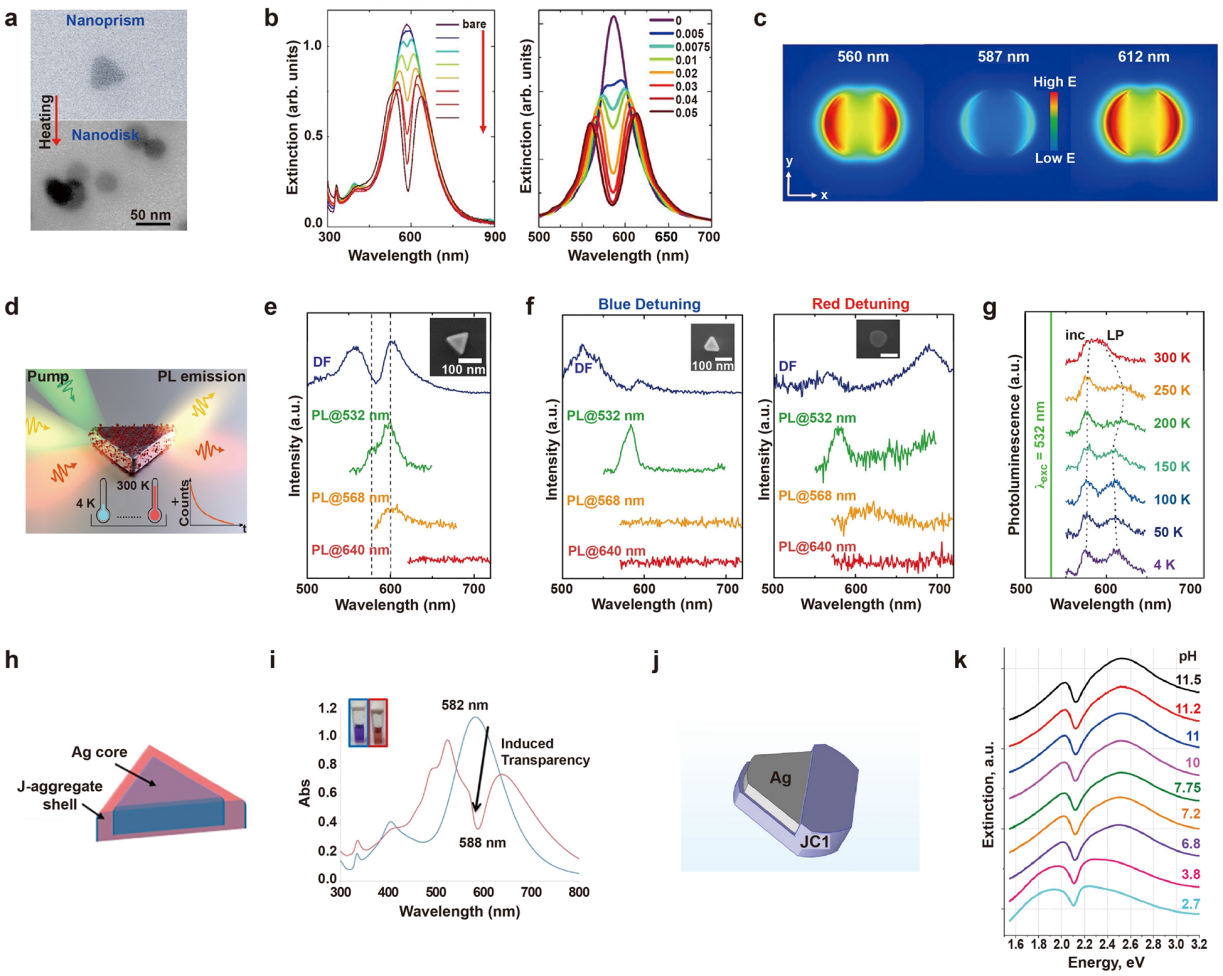
Strong coupling between Ag nanoparticles and J-aggregates. **a** Scanning transmission electron microscope (STEM) images of initial Ag nanoprisms and Ag nanodisks after heating of Ag nanoprisms. **b** (left) Experimental and (right) simulated extinction spectra of Ag nanodisks coupled to the J-aggregates. **c** Electric field distribution of single polaritonic nanoparticle at 560 nm, 587 nm, and 612 nm. **d** Ag nanoprism coupled to the J-aggregates. **e** Scattering and PL spectra of hybrid nanostructures excited by 532, 568, and 640 nm laser excitations. **f** Highly blue-detuned and red-detuned scattering spectra, together with the corresponding PL spectra. **g** Temperature-dependent PL spectra of a single hybrid nanostructure measured at T = 4–300 K. **h** Ag nanoprisms coupled to the J-aggregates. **i** Absorption spectra of Ag nanoprisms@J-aggregates systems. **j** Ag nanoprisms coupled to the J-aggregates. **k** Extinction spectra of Ag nanoprisms@J-aggregates systems at different pH values of the aqueous solution. Reproduced with permissions from [40, 44–46], respectively

Ag is another well-known material in plasmonics due to their low optical loss in the visible and NIR spectral ranges [24]. Disk-shaped nanoparticles offer increased curvature compared to their spherical counterparts, resulting in enhanced localization of the electric field. Furthermore, the presence of flat faces in these nanoparticles makes them advantageous as building blocks for the construction of complex hybrid systems. Balci et al. constructed a hybrid system by coupling Ag nanodisks with TDBC J-aggregates [40]. Compared to Ag nanoprisms, Ag nanodisks offer improved long-term stability, making them a promising candidate for polaritonic applications [41]. The authors synthesized Ag nanoprisms through a seed-mediated method and transformed them into nanodisks via a heating process, altering their LSPR within the visible ranges (Fig. 3a). By adding the TDBC dye solution to Ag nanodisk colloids, J-aggregates were self-assembled onto the nanodisks, resulting in the creation of exciton-polaritons. A large Rabi splitting of 350 meV was observed, indicating successful coupling between plasmons and excitons. The coupling strength between the nanodisk and dye molecules was further modulated by changing the concentration of dye molecules in the reaction medium, which affected the amount of dye molecules adsorbed on the nanodisk. (Fig. 3b, left). FDTD simulations showed good agreement with experimental results when varying the oscillator strength of the J-aggregate (Fig. 3b, right). The large Rabi splitting energy observed in the Ag nanodisks originated from the strong electric field enhancement around the nanoparticle at resonance conditions (~ 587 nm). The electric field intensity of polariton system approached zero at a narrow dip around 587 nm, while bare Ag nanodisk exhibited large electric field localization at the same energy (Fig. 3c), which is also a strong indication of the formation of polaritons.

Ag nanoprisms, in particular, have gained attention because of their easy tuning of resonance frequency and strong electric field enhancement at sharp corners [42, 43]. Wersäll et al. utilized correlative DF scattering and PL spectroscopy to explore polaritonic states composed of Ag nanoprisms and TDBC J-aggregates (Fig. 3d) [44]. They analyzed PL spectra under varying experimental conditions, including different excitation wavelengths, a wide range of plasmon-exciton detunings, and temperatures from 4 to 300 K. In DF spectra, the hybrid structures of Ag nanoprism@TDBC exhibited a Rabi splitting of 200–250 meV. The PL spectra acquired at T = 77 K using three excitation wavelengths (532, 568, and 640 nm), together with the corresponding DF spectra gave distinct spectral features (Fig. 3e). Under 532 nm excitation, the PL spectra displayed two separate peaks associated with emission from uncoupled molecules and incoherent dark states (higher energy peak), as well as emission from the lower polariton state (lower energy peak). At 568 nm excitation, only the lower polariton emission was detected in the PL spectra, while almost no PL signal appeared under 640 nm excitation. The significant spectral overlap between lower energy PL emission peaks and corresponding lower polariton peaks in DF suggest a connection between the lower energy PL peaks and lower polariton emissions. Next, the authors explored the PL spectra from hybrid nanostructures with varying plasmon-exciton detuning (Fig. 3f). In blue-detuned conditions where the plasmon resonance tuned to below the exciton resonance, the lower polariton in PL was less prominent while in red-detuned conditions, the plasmon resonance tuned to above the exciton resonance, the lower polariton state was observable but shifted and broader compared to the DF peak due to phonon-assisted relaxation and radiative processes. Finally, the temperature dependence of the PL spectra excited by a 532 nm laser revealed distinct redshift in the wavelength of polariton peaks at temperatures of 200–250 K, close to the Debye temperature of Ag (Fig. 3g). The results suggested that the lower energy peak in the PL spectra is affected by lower polaritonic nature, while the higher energy peak likely originated from uncoupled molecules and incoherent states, which remain almost temperature-independent. These findings offer new insights into the mechanisms governing relaxation processes in plasmon-exciton hybrid systems. The observed decrease in Rabi splitting in the nanoprism@TDBC system compared to the nanodisk@TDBC system may be attributed to the different distribution of dye molecules in the electric field surrounding the nanoparticles.

DeLacy et al. reported a strong coupling system comprising the same plasmonic nanoparticles, Ag nanoprism, but different J-aggregates, PIC (Fig. 3h) [45]. The authors selected the dimensions and optical properties of the Ag nanoprisms to achieve spectral overlap between the plasmonic resonance and the excitonic resonance of the PIC J-aggregates. Due to the opposite charges, the positively charged PIC dye molecules were electrostatically adsorbed onto the surfaces of the negatively charged of citrate-capped Ag nanoprisms. The absorption spectra exhibited two polariton peaks, a high energy peak and a low energy peak (Fig. 3i). The anticrossing behavior was evident when fitting the peak resonances of the upper and lower branches from the experimental data by adjusting the nanoprism aspect ratio, revealing Rabi splitting energy of 207 meV. The optical characteristics of the hybrid nanocomposites were verified through numerical and analytical methods such as the boundary-element method and temporal coupled-mode theory, respectively. Both theoretical predictions and experimental results confirmed the presence of a transparency dip in hybrid structures, resulting from the strong coupling between the Ag nanoprisms and PIC dyes.

In experiments using the same Ag nanoparticles (AgNPs) but different J-aggregate dyes (TDBC and PIC dyes), a slightly larger Rabi splitting were observed for TDBC dyes. Based on previous research examples, it appears that both PIC and TDBC can facilitate strong coupling in plasmon-exciton hybrid systems, but due to the structural characteristics of TDBC dyes with planar structures, a large number of dyes can be attached to the nanoparticle surface, resulting in larger Rabi splitting as shown in Sect. 2.1.1. Thus, the understanding of J-aggregated structures and their dimensions is crucial for deeper exploration of plasmon-exciton coupling.

Krivenkov et al. utilized other types of J-aggregates, tetrachlorobenzimidazolocarbocyanine (JC-1 or TTC) to investigate the effect of pH on the medium in strong coupling (Fig. 3j) [46]. The main mechanism for the formation of the hybrid system was assumed to involve electrostatic interactions between the anionic groups of sodium citrate (which stabilizes the surface of Ag nanoplates) and the cationic groups of the J-aggregates.

To create a hybrid structure, a JC-1 dye monomer solution was added to the colloidal solution of Ag nanoprisms at pH 11 under stirring. The addition of NaOH, which increases the pH, promoted the formation of J-aggregates. As a result, a J-band at 2.1 eV emerged, coinciding with the maximum of the extinction spectrum of Ag nanoprisms in an aqueous solution. As the concentration of dye monomers increased from 13 to 650 nM, the Rabi splitting increased to 450 meV. To explore the applicability of these hybrid structures for pH sensing, the authors gradually reduced the pH of the aqueous solution by adding HCl and monitored the resulting spectral changes (Fig. 3k). The decrease in pH led to a reduction in Rabi splitting energy from approximately 450 meV at pH 8–11 to about 200 meV at pH 2.5 ± 0.5. The formation of the J-band of JC-1 dyes was found to be pH-dependent, as the shift in the ionic balance of the solution affected the aggregation process of cyanine molecules. Consequently, an excess amount of OH^−^ ions in the solution could alter the ionic equilibrium, causing JC-1 dye to form aggregates with OH^−^. Compared to the similar systems utilizing TDBC and PIC, the Rabi splitting observed here is significantly larger than those two cases. However, the mechanism behind the significant Rabi splitting observed when coupling JC-1 dyes with AgNPs has not been fully elucidated. Obtaining a deeper understanding of the interaction between plasmonic nanoparticles and dye molecules is crucial for understanding the fundamental plasmon-exciton coupling.

#### Bimetallic nanoparticles

AgNPs allow for larger Rabi splitting than AuNPs but the chemical synthesis of AgNPs usually result in a broader size distribution, which affect their optical properties, plasmon resonance wavelengths, and thus, performance in plasmon-exciton interaction studies. Additionally, the shape of the AgNPs discussed so far has been predominantly limited to plate-like shape, as the resonance of isotropic AgNPs shifts to shorter wavelengths compared to the resonance of the J-aggregates. To overcome these limitations, bimetallic nanoparticles are developed. The combination of Au and Ag in nanostructures allows for greater flexibility in designing structures that can match the resonance of J-aggregates, as the resonance of AuNP is shifted to a longer wavelength compared to AgNP of the same shape and size. Thus, the structure, comprising both Au and Ag, facilitates the synthesis of various structures, leveraging the advantages of Ag while preventing surface oxidation.

**Fig. 4:**
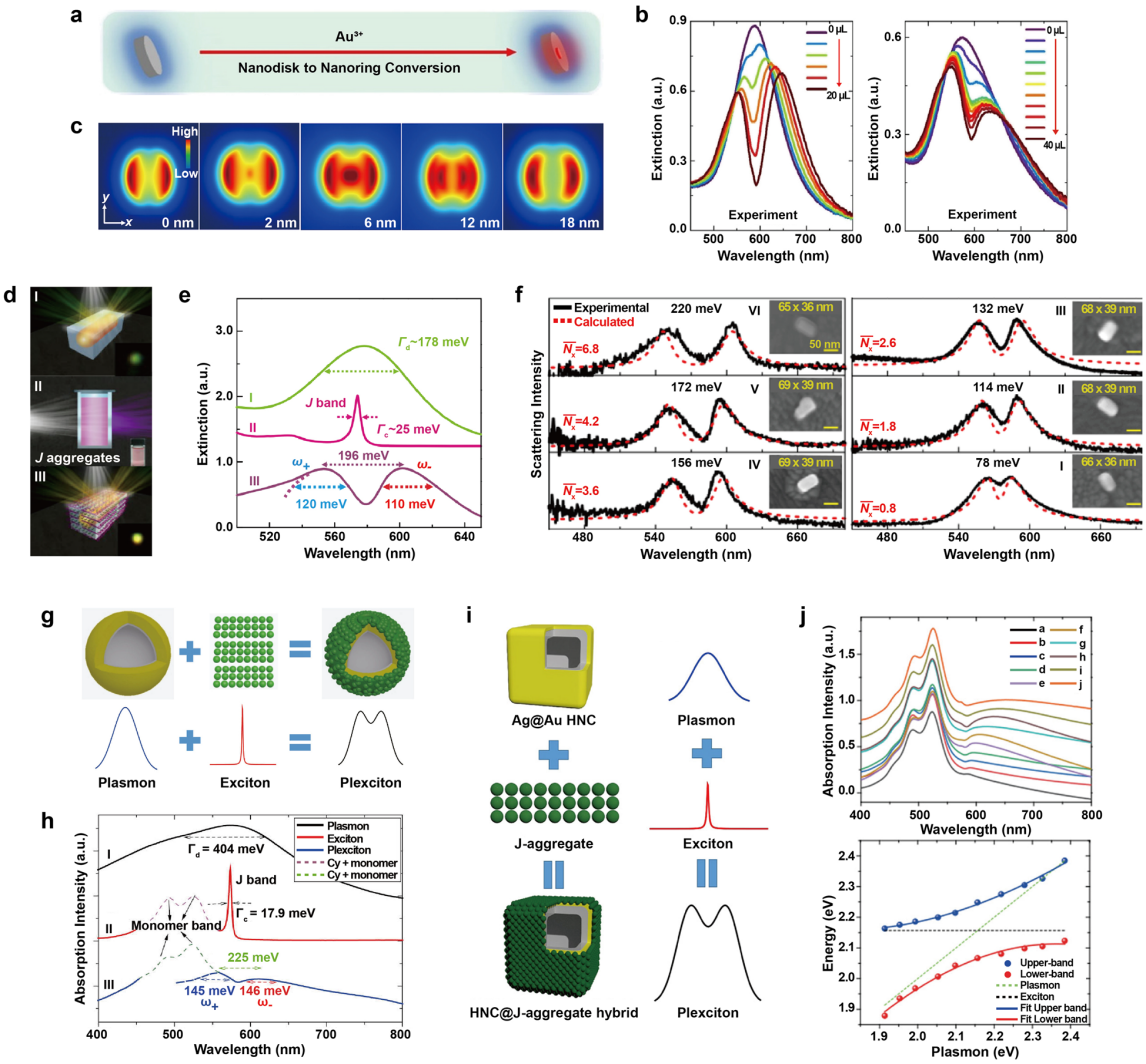
Strong coupling between bimetallic nanoparticles and J-aggregates. **a** Shape engineering of nanodisks into nanorings. **b** Extinction spectra of Ag-Au-alloyed nanorings containing (left) small nanoholes and (right) large nanoholes coupled to the J-aggregates with varying amounts of J-aggregate dye. **c** Electric field distribution of a single nanoring at resonance wavelength with varying inner diameter. **d** Au@Ag nanocuboids coupled to the J-aggregates. **e** Extinction spectra of (I) bare Au@Ag nanocuboids, (II) pristine J-aggregates, and (III) Au@Ag nanocuboid@J-aggregates hybrids. **f** Scattering spectra of the individual hybrids system with different numbers of J-aggregates. **g** Ag@Au HNSs coupled to the J-aggregates. **h** Extinction spectra of (I) bare Ag@Au HNSs, (II) pristine J-aggregates, and (III) Ag@Au HNSs@J-aggregates hybrids. **i** Ag@Au HNCs coupled to the J-aggregates. **j** (top) Extinction spectra and (bottom) anticrossing behavior curves of the Ag@Au HNCs@J-aggregates system. Reproduced with permissions from [47, 49–51], respectively

Guvenc et al. examined the strong coupling of bimetallic plasmonic nanorings with TDBC J-aggregates [47]. Galvanic replacement reactions were employed to synthesize Ag-Au-alloyed nanorings (Fig. 4a). In the presence of Au ions in the solution, Ag atoms on the Ag nanodisk undergo oxidation, leading to the reduction of Au ions near Ag atoms. Importantly, the size of the nanoholes could be effectively controlled by adjusting the amount of Au ions in the reaction medium. By increasing the amount of Au ions in the reaction medium, inner diameter of the nanorings could be enlarged, resulting in tunable plasmon resonance with a shift to a longer wavelength of over 100 nm. When the nanoring colloid was mixed with a TDBC dye solution, the TDBC molecules were able to replace the citrate anions on the surfaces of the nanoparticles and subsequently self-assembled to form J-aggregates. The plasmon resonances of the bimetallic nanorings were then able to strongly couple with the excitonic transitions of the J-aggregates, resulting in a hybridized system with Rabi splitting exceeding 300 meV (Fig. 4b). Numerical simulations with varied inner diameter demonstrated that plasmonic hotspots at 6 nm nanohole diameter exhibited enhanced electromagnetic fields within a central hole (Fig. 4c), providing opportunity to transition the coupling regime from weak to strong via galvanic displacement.

To examine how the plasmon-exciton coupling is influenced by the electric field enhancement and the number of excitons involved, Li et al. demonstrated strong coupling between plasmons confined within a single isolated bimetallic nanoring or nanocuboid and molecular excitons of J-aggregates [48]. The hybrid nanostructures consisted of Au nanoring/nanorod coated with Ag layer (Au@Ag nanoring and Au@Ag nanocuboid) and encapsulated by TDBC J-aggregates. By varying the thickness of the Ag shell, the plasmon resonance of the Au@Ag nanoparticles was tuned crossing the J-aggregates exciton resonance. Extinction and single-particle DF scattering spectroscopy revealed that strong coupling occurred in both Au@Ag nanoring@J-aggregates and Au@Ag nanocuboid@J-aggregates hybrid systems, supporting Rabi splitting of 200 meV for nanorings and 156 meV for nanocuboids, respectively. FDTD simulations demonstrated that the mode volume of the bimetallic nanoring was approximately 2.5 times larger than that of the nanocuboid, with values of 4290 nm^3^ and 1751 nm^3^, respectively. Although the electric field of the nanocuboid was more localized than that of the nanoring, the reduced mode volume of the nanocuboid could lead to weaker coupling strength, as the number of dipoles participating in the coupling would be diminished. FDTD simulations and theoretical calculations revealed that the plasmon-exciton system in an individual Au@Ag nanoring@J-aggregates hybrid has a higher coupling constant (g) of 111.3 meV, involving the number of excitons (N) ~ 196 excitons contributing to coupling, while the Au@Ag nanocuboid@J-aggregates hybrid has ~ 40 excitons contributing to the coupling with g of ~ 87.2 meV. This study illustrates that increasing the overlap volume between excitons and plasmons can result in strong coupling, enabling a larger number of excitons to participate in coupling in the hybrid system. The strength of coupling between plasmonic nanoparticles and excitonic materials can be greatly affected by structural differences in the nanoparticles or the materials. Additionally, the Rabi splitting in Ag-Au-alloyed nanorings increased compared to Au@Ag nanorings, by positioning the dye in a hot spot within a small hole in the Ag-Au-alloyed nanoring structure. These assumptions suggest that the specific morphology of plasmonic nanoparticles with hot spots that generate strong electric fields, plays a crucial role in light-matter interactions. However, it is important to thoroughly investigate other factors, such as the composition ratio and size differences between alloyed and core–shell structures, as they can have a significant impact on the coupling strength.

Liu et al. investigated the strong coupling between similar nanoparticle, Au@Ag nanocuboid, and different J-aggregates, PIC (Fig. 4d) [49]. The LSPR mode of the Au@Ag nanocuboids were aligned with the J-band (approximately 575 nm) of J-aggregated PIC, yielding strongly coupled hybrids that exhibited a mode splitting of around 196 meV (Fig. 4e). The dispersions of these hybrid states displayed distinct anticrossing behavior accompanied by Rabi splitting of approximately 172 meV. When Au@Ag nanocuboids were treated with PIC dye solutions ranging from 0.8 to 8.0 μM, the resulting DF scattering spectra revealed Rabi splitting values between approximately 78 and 220 meV (Fig. 4f). Calculated results suggest that ultrasmall mode volumes (~ 71 nm^3^) of nanocuboid and the short interaction distance between nanocuboids and coated J-aggregates enable achivement of strong coupling in the single J-aggregate exciton levels.

Like the hollow nanoparticle-based strong coupling discussed in the Sect. 2.1.1 and previous examples within this section, Ag@Au hollow nanoshells (HNSs), were also utilized to interact with J-aggregates (Fig. 4g) [50]. The presence of empty space inside hollow structures could lead to enhanced absorption of radiation by utilizing the “light trapping” effect. Typically, solid core nanoparticles can only absorb a small portion of the incoming light. However, in the case of hollow nanoparticles, multiple scattering events can take place inside the empty cavity, leading to an overall improvement in the absorption efficiency of the hollow structure. By adjusting the thickness of Ag shell thickness, the LSPR was tuned to match the resonance of the PIC J-aggregates. The cationic PIC J-aggregates were adsorbed on the surfaces of Cl^−^ ions-modified HNSs through electrostatic interactions, and extinction spectra revealed a Rabi splitting of 225 meV (Fig. 4h). In a follow-up study, the authors investigated the potential of Ag@Au hollow nanocubes (HNCs) as plasmonic nanoparticles for strong coupling, owing to their electric field enhancement at corners and small mode volumes [51]. J-aggregates were coated on Ag@Au HNCs again using the same electrostatic adsorption method mentioned earlier (Fig. 4i). Extinction spectroscopy revealed a Rabi splitting of 179 meV and exhibited anticrossing behavior (Fig. 4j). Although Ag@Au HNCs exhibit a stronger electromagnetic field enhancement compared to Ag@Au HNSs, smaller Rabi splitting observed which is likely due to the smaller interaction area provided by Ag@Au HNCs with the excitonic material.

By combining Ag with Au in core–shell or alloyed structures, it is possible to achieve the benefits of both metals. This approach can lead to improved plasmonic properties, such as large Rabi splitting, as well as increased chemical stability. The Ag-Au hybrid structures can also be designed to have tunable plasmonic properties, making them useful for strong coupling studies.

Regarding the J-aggregates, they possess a combination of desirable properties, including narrow spectral linewidth and large oscillator strength, making them highly suitable for achieving strong coupling with plasmonic nanoparticles. In order to overcome the inherent decay rates of plasmons and achieve efficient light-matter interaction, it is often necessary to incorporate a large number of emitters. In this context, J-aggregates have garnered appreciation due to their ability to form high concentrations on the surfaces of metal nanoparticles. The dense packing of J-aggregates on the nanoparticle surfaces facilitates strong interaction with the plasmonic nanoparticles, leading to enhanced coupling efficiency and improved light-matter interactions. However, accurately determining the precise position of dye molecules attached to plasmonic nanoparticles is challenging. Further investigation into the mechanism of dye molecule adsorption and a deeper exploration of the strong coupling between plasmons and dye molecules would be valuable.

### Coupling with 2D transition metal dichalcogenides

2D transition metal dichalcogenides (TMDCs), such as MoS_2_, WSe_2_, and WS_2_ have emerged as a promising alternative to J-aggregates for achieving strong coupling between plasmons and excitons [52–55]. When reduced to monolayers, these semiconductor nanosheets transform into direct band-gap materials, accommodating excitons with exceptionally large binding energy and high oscillator strength [56, 57]. This is due to the intense Coulomb interaction and diminished dielectric screening in atomically thin structures. Consequently, excitons in TMDC monolayers remain tightly bound even at room temperature, resulting in robust light absorption and PL. This enables strong coupling between surface plasmons in compact nanocavities and excitons in 2D materials that can function at room temperature.

#### Au nanoparticles

**Fig. 5:**
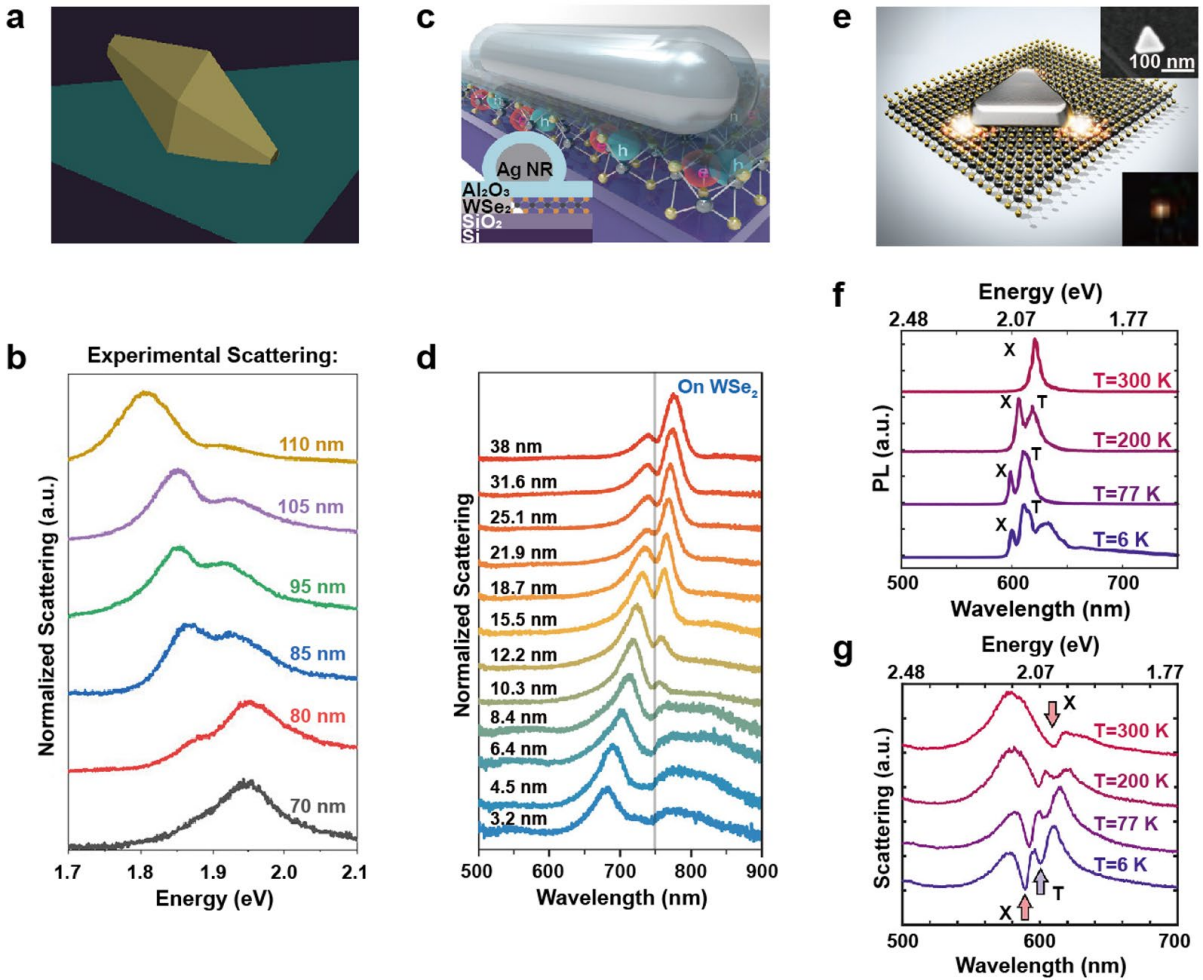
Strong coupling between plasmonic nanoparticles and transition metal dichalcogenides. **a** AuBPs coupled to the MoS_2_ monolayer. **b** Scattering spectra of AuBPs/MoS_2_ hybrids with varying lengths of AuBPs. **c** AgNRs coupled to the WSe_2_ monolayer. **d** Scattering spectra of AgNR/WSe_2_ hybrids with varying alumina coating. **e** Ag nanoprisms coupled to the WS_2_ monolayer. **f** PL spectra of the WS_2_ as a function of temperature. **g** Scattering spectra of Ag nanoprism/WS_2_ hybrids at T = 300, 200, 77, and 6 K. Reproduced with permissions from [52, 53, 55], respectively

Lawless et al. investigated the interaction between AuBPs and a MoS_2_ monolayer [52]. The AuBPs offer several benefits, such as a sharp tip for localizing and enhancing the electric field and easily adjustable longitudinal plasmon resonance via manipulation of dimensions like size, length, tip radius, and aspect ratio. MoS_2_ was used for coupling with the bipyramid, taking advantage of its unique electrical and optical properties [58, 59]. The transition from an indirect to a direct band-gap in the monolayer configuration results in a significant enhancement of light emission and optical absorption [57]. Moreover, the use of MoS_2_ provides additional advantages such as a relatively low synthesis temperature [60] and an exciton energy within the visible range [61]. The hybrid bipyramid/MoS_2_ structure was fabricated by drop casting synthesized AuBPs onto monolayered MoS_2_ flakes (Fig. 5a). As a consequence of the coupling between the longitudinal plasmon mode and the exciton mode, Rabi splitting was observed in the scattering spectrum of a single AuBPs placed on the monolayered MoS_2_ substrate, as the length of the bipyramid was varied between 70 and 110 nm with a similar aspect ratio of ~ 2.3 (Fig. 5b). As bipyramid lengths increased, the Rabi splitting increased from ~ 55 meV for 70-nm long bipyramids to ~ 80 meV for 100-nm long bipyramids. FDTD simulated spectra also exhibited two polariton peaks and a central dip corresponding to the exciton energy. The coupling strength was strongly influenced by both the tip radius and the length of the bipyramid. To achieve optimal field enhancements and Rabi splitting, sharp tips with radii less than 7 nm were required for bipyramids maintaining a fixed aspect ratio. The field strength was found to be highly dependent on bipyramid length, with larger bipyramids being favored for robust coupling due to the field enhancement being localized at the tips. Additionally, the tip sharpness played a crucial role, and if it was not sufficient, a transition to the weak coupling regime occurred.

#### Ag nanoparticles

WSe_2_ is known for its high absorption coefficient in the visible to infrared range, as well as its high quantum yield in PL and strong spin–orbit coupling [62, 63]. The first report of strong coupling between excitons in a single atomic layer of WSe_2_ and plasmons in Ag nanorod (AgNR) was reported by Zheng et al. (Fig. 5c) [53]. They specifically chose the multipolar plasmon resonance (n = 3) due to its superior quality factor (Q) in comparison to the dipolar plasmon resonance (n = 1) [64]. The former exhibited a Q value approximately three times higher and a narrow resonance linewidth of 57 meV. The authors deposited a 3.2 nm alumina layer onto the WSe_2_ as a spacer to prevent direct nanoparticle contact and eliminate potential charge transfer processes. As the refractive index of alumina is higher than that of air, the surface plasmon energy experiences a gradual redshift due to the dielectric screening effect. In the case of a nanorod on a substrate without WSe_2_, the plasmon resonance redshifted from 646 to 735 nm as the coating thickness increased from 3.2 to 38 nm. The coupling of the nanorod on WSe_2_ led in mode splitting in the single-particle scattering spectra, exhibiting a clear anticrossing behavior with a Rabi splitting of 49.5 meV (Fig. 5d). The calculated scattering spectra for a single nanorod with WSe_2_ displayed two scattering peaks, consistent with the experimental findings. Additionally, the number of excitons (N) contributing to the polariton state was estimated to be N = 4100. The coupling rate of each individual exciton at position r to the plasmon is initially calculated quantifying the strength of interaction between the exciton and the plasmon at that specific location. Subsequently, the contributions from different excitons are summed by performing an integral of g(r), which accounts for the coupling strength across the entire system. By adjusting the exciton density, the maximum value of coupling strength could be tuned to match the experimentally measured g value of 24.75 meV. Detailed explanation of the calculation method can be found in the Supporting Information of the paper [53].

Cuadra et al. coupled a WS_2_ monolayer with Ag nanoprisms to take advantage of the strong local electric field at the corners of the nanoprisms (Fig. 5e) [55]. WS_2_ can support both neutral (X) and charged (T) exciton resonances. PL analysis confirmed these resonances, which could be modulated by altering the system's temperature (Fig. 5f). At 300 K, the PL spectrum displayed a maximum at 2.012 eV, corresponding to the neutral exciton with a binding energy of around 700 meV. At 200 K and 77 K, two peaks emerged in the PL spectrum at energies of approximately 2.07 eV and 2.03 eV, corresponding to the neutral exciton and positively charged exciton (trion), respectively. At 6 K, additional peaks were observed in the PL spectrum, with the two high-energy peaks representing the exciton and trion, whereas the additional peaks may arise due to bound excitons. The authors then constructed the hybrid system by placing Ag nanoprisms on the WS_2_ monolayer through drop casting a nanoparticle suspension onto a polymer-coated substrate. At room temperature, scattering spectrum showed mode splitting with a Rabi splitting of ~ 120 meV, due to the strong coupling between the plasmon and the exciton in WS_2_. As the temperature decreased, trions contributed more prominently to the PL spectrum, leading to two dips in the DF scattering spectrum (Fig. 5g). The Rabi splitting between the upper and lower polariton branches was approximately 150 meV, which is ~ 30 meV larger than the Rabi splitting at room temperature due to the narrowing of both plasmon resonance and exciton resonance linewidths upon cooling. These findings demonstrate the potential for exploring electrically charged polaritons in the form of plasmon-exciton-trion hybrids.

In comparison to organic J-aggregates, the Rabi splitting observed in TMDCs is generally weaker. In TMDC-based hybrid systems, the interaction between plasmons and excitons is limited to the contact area between the TMDC layer and plasmonic nanoparticles, whereas J-aggregates can coat the entire surface of plasmonic nanoparticles, providing increased opportunities for interaction between two components. The design of nanoparticles with flat surfaces enables increased interaction area and stronger electric fields at edges, which would be beneficial for enhancing light-material interactions and achieving superior performance in TMDC-based polariton systems.

### Coupling with quantum dots

Colloidal quantum dots (QDs) are semiconductor nanocrystals that exhibit unique optical and electronic properties resulting from the quantum confinement effect. QDs offer size-dependent optical properties, making them highly versatile for various applications [65]. QDs offer several advantages over molecular systems, including a broad absorption spectrum and relatively narrow emission bands [66].

**Fig. 6:**
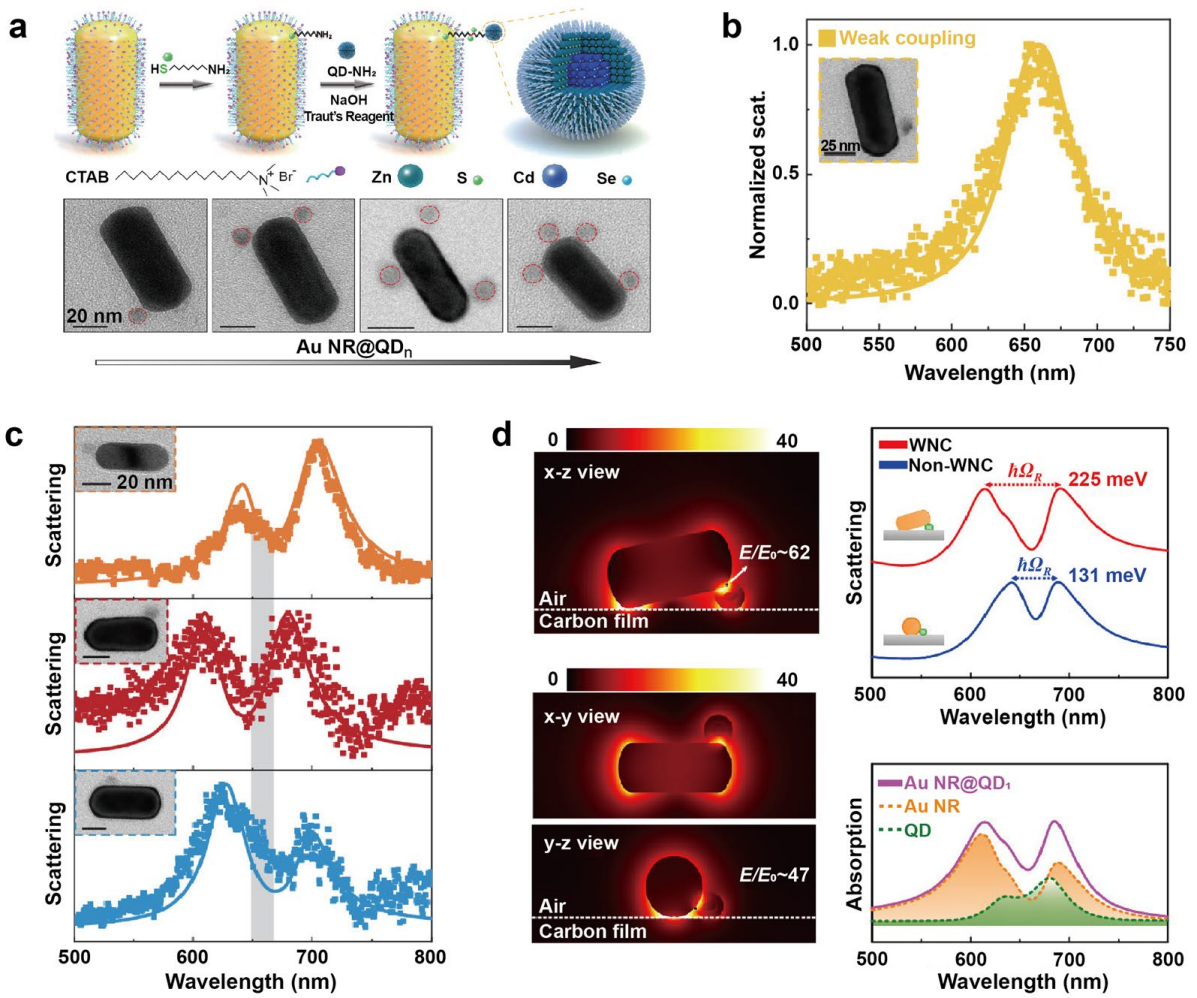
Strong coupling between Au nanorod and quantum dot. **a** Schematic of the assembly process for the AuNR@QD_*n*_ hybrids and transmission electron microscope (TEM) images for typical AuNR@QD_*n*_ hybrids integrated with *n* = 1, 2, 3, and 4 QDs, respectively. **b** A weak coupling of AuNR@QD with a distance of ∼2 nm between the nanorod and QD. **c** Normalized scattering spectra of three individual AuNR@QD hybrids with different detuning. **d** (left) Simulated electric field distributions of hybrid WNC system and (right) scattering spectra of the hybrid WNC and non-WNC systems. Reproduced with permissions from [67]

Li et al. reported strong coupling between a QD and a single AuNR by employing surface functionalization with organic molecules [67]. To demonstrate a single QD strong coupling system, AuNR@QD hybrids were fabricated using a self-assembly method, with 11-Amino-1-undecanethiol hydrochloride as the linker molecule. By varying the concentration of cetyltrimethylammonium bromide (CTAB) molecules, it was possible to control the number of QDs on individual AuNR (Fig. 6a). The AuNR@QD hybrids were then dropped onto a carbon film substrate and the integrated QD was pressed below or attached sideways to the AuNR, confining the distance between the two components to a range of 0 to 2 nm randomly. For hybrids with a distance of ~ 2 nm between the nanorod and QD, the coupling was found to be in the weak coupling regime (Fig. 6b). When a QD was positioned below one end of a nanorod and contacted the nanorod via a crystal face, it led to the formation of a wedge nanogap cavity (WNC) between the QD and nanorod, which effectively enhanced the electric field and yield a substantial Rabi splitting of ~ 234 meV with a coupling strength g of up to ~ 114.34 meV (Fig. 6c). Numerical simulations showed large Rabi splitting of ~ 225 meV and ~ 131 meV for WNC and non-WNC configurations, respectively, which were in good agreement with the measured Rabi splitting of ~ 234 meV and ~ 121 meV (Fig. 6d). Through the process of dropping the AuNR@QD hybrids onto a carbon film substrate, the QDs are brought into contact with the AuNR facets, resulting in spatial proximity between the two components. This positioning places the QD in the region of maximum electric field of the single AuNRs, resulting in strong coupling with large Rabi splitting, even with only one QD attached.

While strong coupling between plasmonic nanoparticles and QDs hasn't received as much attention as the emitters discussed in previous sections, it offers a significant potential for exploration. The number of QDs interacting with plasmonic nanoparticles is typically fewer due to their larger size. Thus, to overcome this limitation, the design of plasmonic nanoparticles, specifically to enhance electric field confinement and localization, could foster strong coupling of QDs even within colloidal systems.

### Coupling with perovskites

Lead halide perovskites (LHPs) have garnered significant attention as active materials in photovoltaics and light-emitting diodes due to their strong absorption [68], low trap densities [69], high oscillator strengths [70], large exciton binding energies [71], and narrow linewidths. The ability to synthesize LHPs with varying dimensionalities has made low-dimensional perovskites an attractive option for tailoring interactions between light and matter [72].

**Fig. 7:**
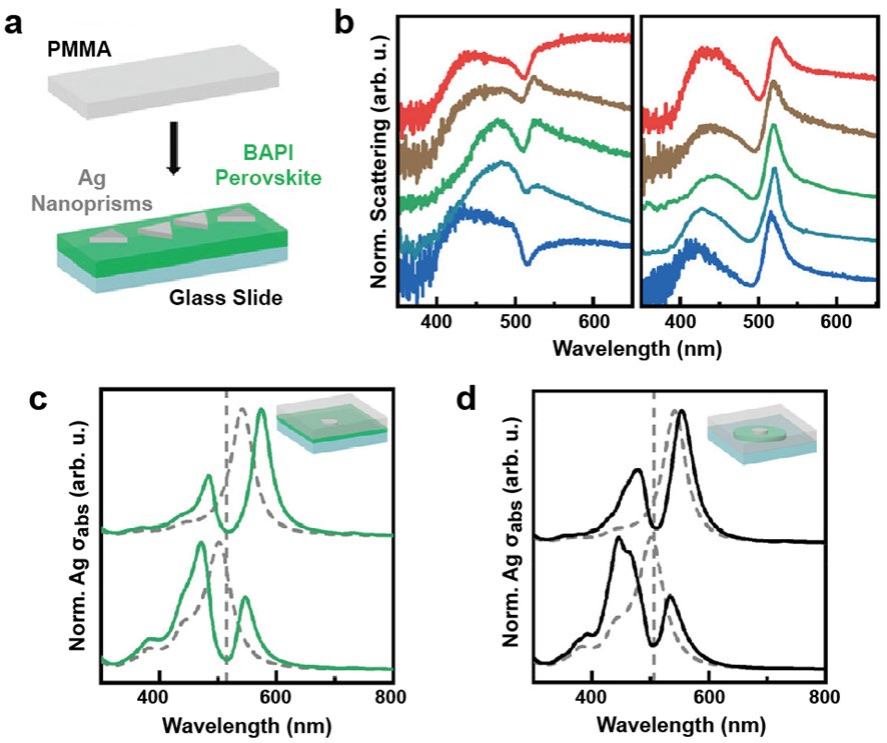
Strong coupling between Ag nanoprisms and perovskites. **a** AgNP coupled to 2D BAPI perovskites. **b** Scattering spectra of the individual hybrid system. Simulated absorption spectra of the Ag nanodisks with different sizes coupled to **c** 2D BAPI and **d** BAPI grain. Reproduced with permissions from [72]

Muckel et al. reported strong coupling with a Rabi splitting of ~350 meV in a system that combined LHPs and Ag nanoprisms ranging in size from 15 to 50 nm, with plasmon resonances centered around 480–520 nm [72]. Separately, (C_4_H_9_NH_3_)_2_PbI_4_ (BAPI) perovskite film was prepared using a spin coating method. The synthesized Ag nanoprisms were then transferred from an aqueous solution to hexane and applied via spin coating onto BAPI perovskite film, followed by the application of a PMMA top layer (Fig. 7a). DF scattering spectra showed individual nanoparticles coupled to the BAPI film and displayed two peaks with a dip between 490 and 515 nm, associated with the BAPI excitonic transition or hybrid Fano resonance (Fig. 7b). Although mode splitting in scattering can occur in two regimes—Fano interaction or Rabi splitting—FDTD simulations of coupled systems were conducted since mode splitting in absorption signals typically represents strong coupling more clearly. When simulating the system, authors simplified nanoprisms as nanodisks, neglected the 2D perovskite microstructure and modeled BAPI as a continuous layer. The resulting spectra showed a splitting with a dip at around 513 nm (Fig. 7c). The simulated absorption spectra of Ag nanodisk coupled to a BAPI grain also showed a splitting pattern, with a significant dip (Fig. 7d). The strength of coupling in the system was determined by analyzing the spectral peak positions in the simulated absorption spectra and it revealed a Rabi splitting of 350 meV, which satisfies the criteria for strong coupling (Ω > γ_p_, γ_2D_ where γ_P_ = 202 ± 11 meV, γ_2D_ = 55 ± 11 meV).

Although perovskite exhibits excellent optoelectronic properties, one of its drawbacks is its susceptibility to degradation in the presence of highly polar solvents, such as water. When plasmonic nanoparticles synthesized in water are transferred to an organic solvent, post-processing steps are required to ensure their stability and functionality in the organic solvent. If it is feasible to synthesize stable perovskite materials capable of enduring long-term exposure to water, their potential for practical applications would be significantly enhanced.

### Biexcitonic system

Similar to the single-photon process, two-photon Rabi oscillation occurs when the exchange rate of two photons between an emitter and an electromagnetic field surpasses their decay rates, offering a foundation for multiphoton coherent control [73, 74]. The emergence of multiple Rabi splitting in such systems is of interest to both fundamental and applied sciences, offering opportunities to investigate multimode hybridization and energy transfer [75–77].

#### Two different J-aggregates

Melnikau et al. revealed unique multiple spectral features by developing multicomponent systems comprising core–shell Au@Ag nanocuboids and two J-aggregated cyanine dyes [78]. J-aggregates of JC-1 and 2-[3-[1,1-dimethyl-3-(4-sulfobutyl)-1,3-dihydro-benzo[e]indol-2-ylidene]-propenyl]-1,1-dimethyl-3-(4-sulfobutyl)-1H-benzo[e]indolium hydroxide (DBI) dyes were used, exhibiting narrow peaks at 587 and 637 nm for JC-1 and DBI dyes, respectively. J-aggregates of the DBI dyes did not form a bound complex with bare Au@Ag nanocuboids due to its specific chemical structure and polarity. However, a multilayer hybrid system consisting of DBI J-aggregates and nanocuboid@JC-1 structure was formed, facilitated by the presence of an oppositely charged layer of JC-1 J-aggregates on the nanoparticle surface.

**Fig. 8:**
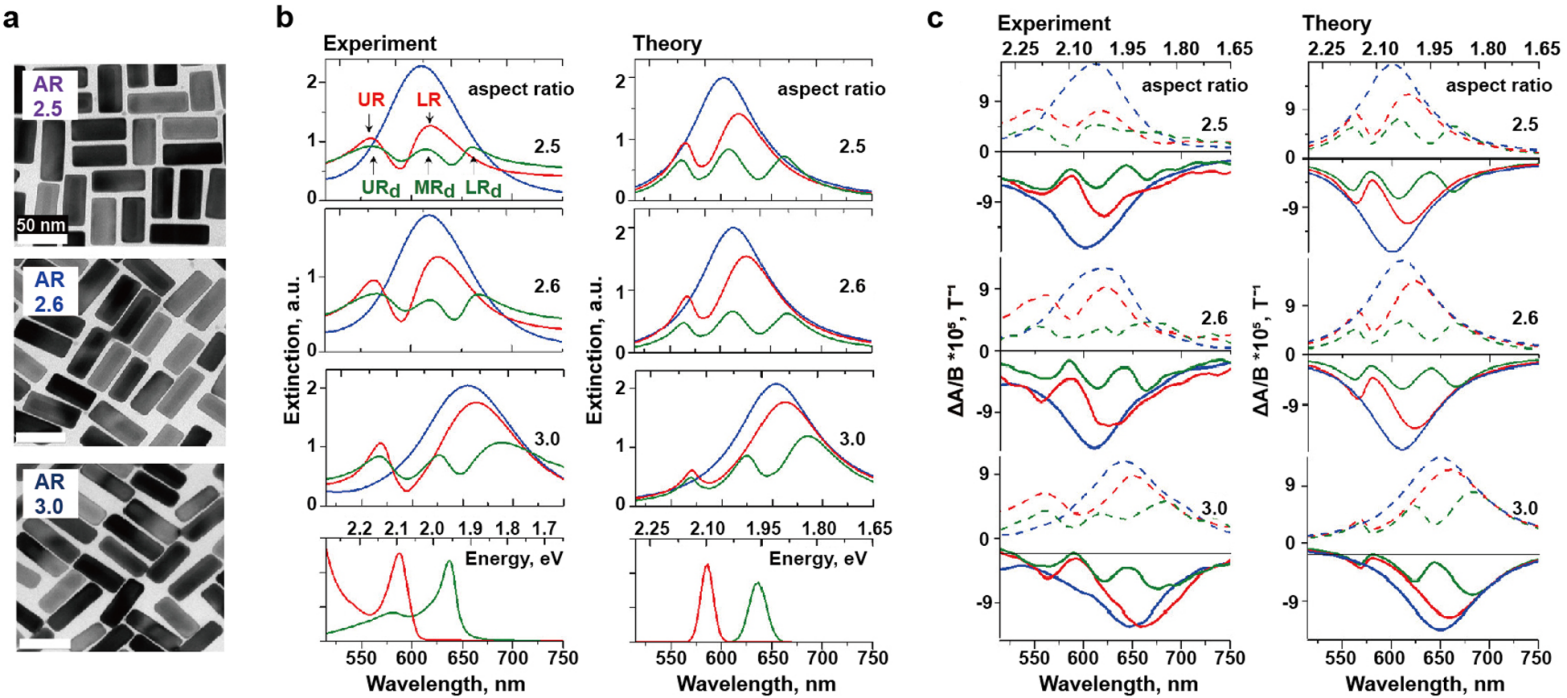
Strong coupling between bimetallic nanoparticles and two J-aggregates. **a** TEM images of Au@Ag nanocuboids. Experimental and theoretical **b** extinction and **c** MCD spectra of (blue) bare Au@Ag nanocuboids, (red) Au@Ag nanocuboids coupled to the J-aggregates of JC-1, and Au@Ag nanocuboids coupled to the J-aggregates of two fluorophores (JC-1 and DBI), with different aspect ratios. Reproduced with permissions from [78]

By adjusting the thickness of the Ag shell deposited on the surfaces of the AuNR core, the aspect ratio can be varied from 3 to 2.5 (Fig. 8a), shifting the longitudinal LSPR from 655 to 610 nm to overlap the spectral positions of the J-band of the two dyes and the plasmon resonance. Then, the driving force for coating Au@Ag nanocuboid with two different layers of J-aggregates was the interaction between dyes with opposite charges; JC-1 with cationic groups and DBI with anionic groups. The electrostatic interaction between the cationic stabilizing agent of Au@Ag nanocuboid and the anionic groups of JC-1 J-aggregates resulted in the adsorption of JC-1 J-aggregates onto the nanocuboid surfaces first [79]. Subsequently, the DBI J-aggregates were coated on the Au@Ag nanocuboid@JC-1, forming a multicomponent hybrid system. The extinction spectrum verified the occurrence of strong coupling with double Rabi splitting in the multicomponent polariton nanostructures, both experimentally and theoretically (Fig. 8b). In the extinction spectrum of Au@Ag nanocuboid@JC-1, two polariton peaks, upper resonance and lower resonance, appear with a large Rabi splitting of 175 meV. The interaction between the lower resonance of the Au@Ag nanocuboid@JC-1 and the J-band of the DBI dyes then resulted in a new multicomponent structure with double Rabi splitting, with Rabi splitting values of 175 and 163 meV. Interestingly, the authors found strong magneto-optical activity in multicomponent polariton nanostructures (Fig. 8c). In the hybrid structures of nanocuboid@JC-1@DBI, the magnetic circular dichroism (MCD) spectra were well-aligned with the absorption spectra. The MCD spectra indicated that the three polariton states exhibited significant magnetic properties, which could be attributed to the interactions in the strong coupling regime between the J-aggregates of the two nonmagnetic dyes and the plasmonic nanostructure. These findings may produce a significant development for new sensing systems based on magneto-optical activity.

#### J-aggregates and WS_2_

Different type of excitonic properties, a 2D material and dye molecule, can be simultaneously coupled to plasmonic nanoparticles. Zhang et al. reported a biexcitonic strong coupling system by integrating two different excitonic properties, a TMDC monolayer and J-aggregates, on AuNCs [80]. WS_2_ monolayer and PIC J-aggregates were chosen as exciton materials due to their heterogeneity resulting from distinct morphologies and characteristics, with a large exciton energy detuning of about five times (γ_PIC_ = 25 meV and γ_WS2_ = 145 meV).

**Fig. 9:**
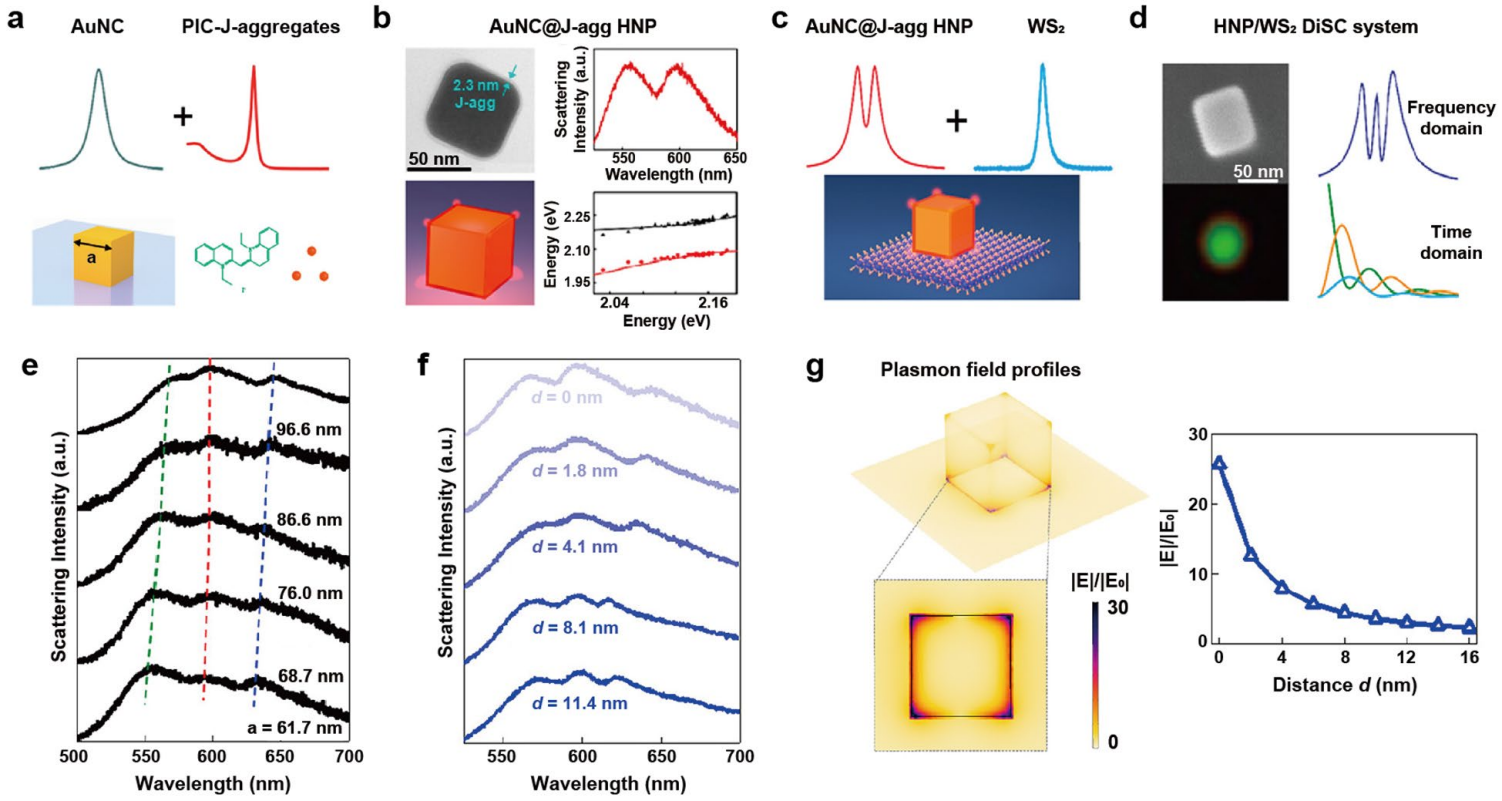
Strong coupling between AuNCs and two different excitonic materials. **a** AuNC coupled to the J-aggregates. **b** Experimentally measured scattering spectra and anticrossing behavior curves of AuNC@J-aggregates. **c** AuNC@J-aggregates coupled to the layer of WS_2_. **d** Three-peak spectrum and the temporal dynamics of AuNC@J-agg/WS_2_ hybrids. Scattering spectra of individual AuNC@J-aggregates/WS_2_ hybrids with **e** varying AuNC sizes and **f** varying the thickness of PMMA between the AuNC@J-agg and the WS_2_. **g** Numerical simulated field enhancement of the AuNC@J-aggregates/WS_2_ system varying the thickness of spacer. Reproduced with permissions from [80]

First, the authors constructed a polariton system that coupled plasmons with single excitons by coating AuNC with PIC J-aggregates using electrostatic self-assembly (Fig. 9a). Single-particle DF scattering measurements revealed mode splitting in AuNC@J-aggregates system with a ~ 2 nm layer of J-aggregates, and the coupling strength was determined to be 90 meV (Fig. 9b). Subsequently, AuNC@J-aggregates were transferred to the surface of WS_2_ monolayer (Fig. 9c and d). Scattering spectra of five AuNC@J-aggregates/WS_2_ with varying AuNC sizes exhibited three scattering peaks corresponding to the upper polariton, middle polariton, and lower polariton branches of the biexcitonic strong coupling nanosystem (Fig. 9e), with a coupling strength of 90 meV for each.

Moreover, introducing a poly(methyl methacrylate) (PMMA) spacer between the AuNC@J-aggregates and the WS_2_ monolayer facilitated manipulation of the coupling strength. When the spacer thickness exceeded 8 nm, the WS_2_ exciton became isolated and effectively decoupled from the AuNC@J-aggregates (Fig. 9f). As the spacer thickness increased, numerical simulations revealed a reduction in field enhancement at the WS_2_ surface beneath the cube corners (Fig. 9g). This decrease explained the observed decoupling between the AuNC@J-aggregates hybrid nanoparticle and WS_2_, in line with the energy shift of the lower polariton. The examination of the biexcitonic strong coupling system not only elucidate the simultaneous coupling behaviors of two different excitons but also offer a platform with enhanced degrees of freedom to study quantum mechanics, potentially inspiring the design of future room-temperature quantum devices and quantum networks.

In summary, the Rabi splitting in single nanoparticles exhibits a broad range of values (55–450 meV), illustrating the interactions between various plasmonic nanostructures and excitonic systems, including J-aggregates, TMDCs, QDs, and perovskites. The observed differences in the coupling strength can be attributed to several factors, including the composition, size, and shape of the nanoparticles, as well as the number and the properties of excitons involved in the strong coupling. Given the limitations associated with generating strong electric field within a single nanostructure, it would be important to develop systems that can effectively interact with a substantial population of excitons.

## Strong coupling in nanoparticle dimers

Single colloidal nanoparticles successfully have shown the plasmon-exciton coupling, however, these structures exhibit limited electric field confinement and relatively large mode volumes. Consequently, nanoparticles containing small gaps, which support gap plasmons, have been applied to further explore the plasmon-exciton interaction in a strong coupling regime. In light-matter interactions, the presence of metal nanogaps is recognized for its ability to concentrate incident light. The extremely small distances in nanogaps can amplify the local electromagnetic field intensity by several orders of magnitude, leading to significantly smaller mode volumes compared to individual nanoparticles. The strong confinement of light at junction areas, commonly referred to as “hotspots,” greatly enhances light-matter interactions, making nanogaps a critical feature in various plasmonic applications such as SERS for chemical sensing, plasmon-enhanced spectroscopy, and nanophotonic devices. The most basic structure that exhibits nanogaps is a dimer, consisting of two metallic particles joined together as a single unit. In this section, we present two examples of strong coupling in nanoparticle dimer systems and explore how this coupling was achieved using only a minimal number of molecular emitters. Subsequently, in Sect. 4, we will delve into the discussion of nanogaps formed between a nanoparticle and a 2D surface.

There are three key factors to use dimers for strong coupling; (i) robust assembly of plasmonic nanocavities with reliable nanogaps (d < 5 nm), (ii) tunable nanogap distances to modify the coupling strength, and (iii) precise placement of single or multiple emitters into such cavities with a high degree of spatial control. Forming a sub-10 nm gap with an accuracy of ~ 1 nm in nanostructures is still challenging for top-down approaches such as electron beam lithography (EBL) technique. A bottom-up assembly, such as DNA conjugation [81], in contrast, allows for precise control over inter-particle separation. Self-assembly method also offers a means to incorporate emitters of interest such as fluorophores and Raman molecules in chemically designed bridging linkers.

Despite these advances, nanoparticle dimer formation still faces low-yield and scalability challenges. The polarization of incident light also plays a crucial role in polariton formation in nanoparticle dimers. An earlier study demonstrated that when J-aggregates of a cyanine dye were placed on top of lithographically fabricated nanoparticle dimers, a split scattering spectrum was observed only upon excitation with light polarized in the longitudinal direction, not with light polarized in the transverse direction [82]. This polarization-dependent coupling phenomenon, along with relatively low yields, necessitates the characterization of strong coupling with dimeric structures specifically at a single-particle level using DF microscopy. Contrary to ensemble measurements, single-particle analysis focuses on individual nanoparticle behavior and is ideal for characterizing the unique properties of individual particles since this approach minimizes the impact of sample heterogeneity.

### Coupling with J-aggregates

In the first example, the authors employed DNA origami templates to construct nanoparticle dimers for plasmon-exciton coupling [16]. The DNA origami technique involves utilizing a single strand of DNA, typically from a virus, as a scaffold to create nanoscale structures with accurate, programmable designs. Complementary short synthetic DNA sequences, referred to as “staple strands,” are used to fold the DNA scaffold into a specific shape. When nanoparticles are conjugated to these staple DNAs, they adopt a configuration determined by the DNA scaffold. This method provides precise control over the size and shape of the resulting nanoparticle assemblies, allowing the design of metallic structures that influence the optical properties of nearby components, such as customizing hot spots for SERS, modulating fluorophore fluorescence, and controlling colloidal quantum dot properties [83–87].

**Fig. 10:**
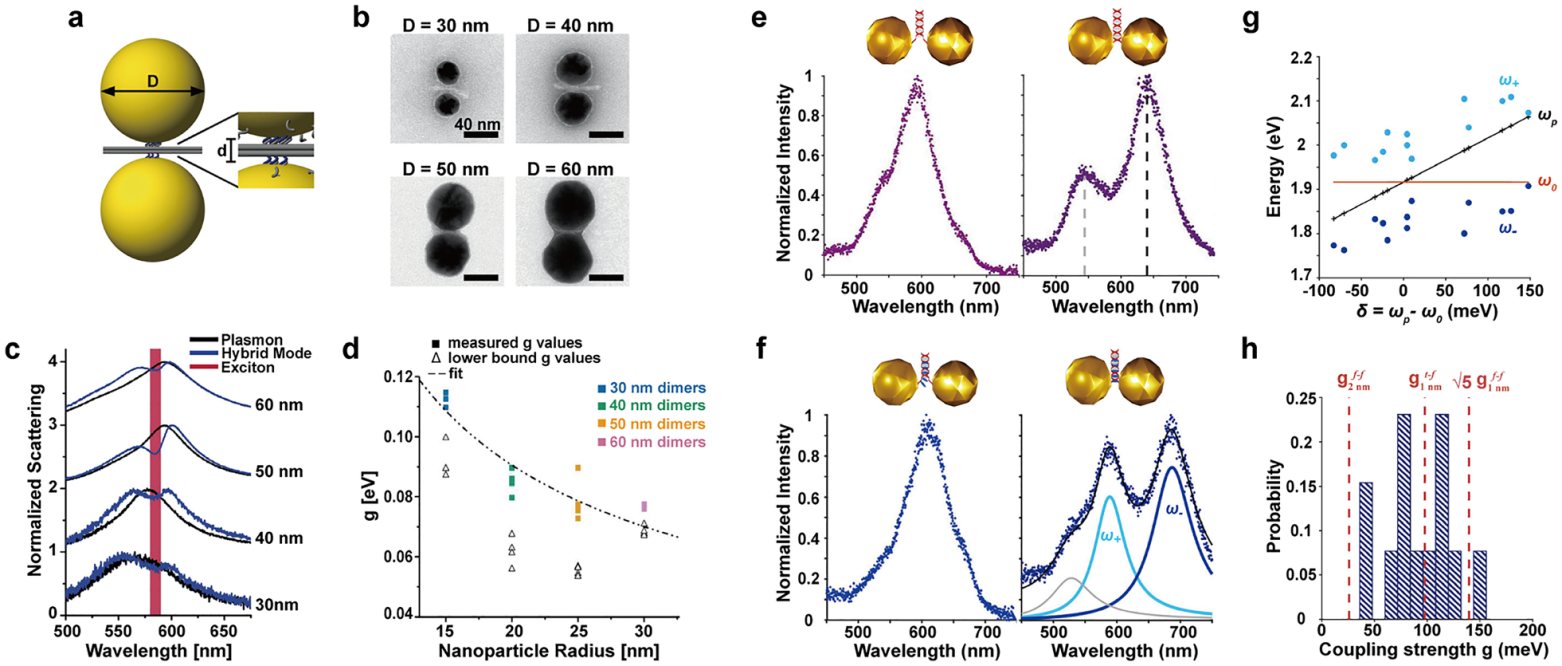
Coupling between Au nanoparticle dimers and molecular emitters. **a** Schematic of two layers of DNA origami sheet templating AuNP dimer. **b** TEM images of AuNPs accommodated by DNA sheets. **c** Normalized scattering spectra before and after photobleaching the J-aggregates. **d** Correlation function between the coupling strength and particle radius. **e** 40 nm AuNP dimers attached by DNA double-strand and single nanostructure resonance spectra for different ionic strengths, (left) 250 mM and (right) 1 M. **f** 40 nm AuNP dimers attached by DNA double-strand featuring Atto647N molecules and scattering spectra for different ionic strengths, (left) 250 mM and (right) 1 M. **g** Distribution of ω_±_ as a function of energy detuning, σ = ω_p_ - ω_0_. **h** Distribution of estimated coupling strength in comparison to the simulated values. Reproduced with permissions from [16, 88], respectively

Here, the origami was designed to assemble AuNPs into a dimer with a separation of approximately 5 nm (Fig. 10a). Initially, the AuNPs-origami mixture was incubated, followed by dimer extraction through gel electrophoresis. The dimer structures were then deposited onto glass slides and immersed in a bath containing cyanine-based dyes that readily form J-aggregates in water. Notably, excitons were incorporated not only at the hotspots but also randomly around the dimers. By varying the nanoparticle diameter from 30, 40, 50, to 60 nm (Fig. 10b), the plasmon resonance could be tuned across the energy frequency of a J-aggregate exciton. The spectral overlap between plasmons and excitons led to strong plasmon-exciton coupling and mode splitting in their single-particle scattering spectra (Fig. 10c). The plasmon resonance of structures without exciton contributions was measured by exposing samples to continuous white light illumination for 1 h to bleach the J-aggregates. Although the spectral shape did not distinctly reveal well-resolved transverse and longitudinal peaks, diameter-dependent splitting occurred. From the position and linewidth of the excitons (ω_qe_ = 2.14 eV, Γ_qe_ = 30 meV) and plasmons (ω_p_ = 2.14 eV, Γ_p,avg_ = 230 meV), the g was estimated to be ~ 90 meV, resulting in a Rabi splitting of ~ 150 meV. A correlation function between g and particle radius, R (g ~ 1/R^n^ with n = 0.63), was also developed due to the stronger coupling observed in smaller particles (Fig. 10d). However, for smaller nanoparticle systems, scattering decreased sharply, and surface scattering damping was expected to become dominant, making it difficult to measure signals from particles smaller than 30 nm.

Although this study utilized dimerically assembled structures for strong coupling, it remains unclear whether the authors explicitly and exclusively benefitted from field confinement arising from closely spaced metallic nanocrystals. Given that the DNA origami technique enables precise incorporation and positioning of additional nanocomponents, it would be advantageous to accurately position individual components, such as dyes or quantum dots, in hotspots and investigate polariton formation.

### Coupling with dye molecules

Simple hybridization between two DNA strands could be also employed to build nanoparticle dimers while providing active control over both the number and position of non-aggregated dye molecules at the nanoscale within a dimer. By reaching sub-2 nm interparticle spacings in a controlled way, Heintz et al. realized strong coupling with a limited number of quantum emitters [88]. In this study, the authors utilized a short (50 bp) DNA double-strand to produce 40 nm AuNP dimers in a 80% yield. As electrostatic repulsion between particles hindered close assembly with interparticle distances below 10 nm, the authors increased the local ionic strength to 250 mM and eventually to 1 M. At an intermediate ionic strength of 250 mM, plasmon resonance spectra displayed a primary longitudinal mode with a minor red shift and a transverse mode appearing as a shoulder (Fig. 10e, left). At a high ionic strength, plasmon resonance spectra exhibited a red-shifted longitudinal mode and a transverse mode overlapping with a quadrupolar longitudinal mode (Fig. 10e, right).

To incorporate five dye molecules in the gap between 40 nm AuNPs, the authors employed a DNA strand with five cytosine bases functionalized by an Atto647N molecule (ω_0_ = 1.916 eV, 647 nm), selected because their absorption spectrum peaks align with the average longitudinal resonance wavelength. The resulting hybrid structures demonstrated strongly coupled modes in the single-particle scattering spectra when interparticle distances decreased below 2 nm (Fig. 10f). The distribution of hybrid wavelengths as a function of energy detuning clearly exhibited the anticrossing behavior characteristic of strong coupling in the visible range (Fig. 10g). The measured coupling strengths ranged from 50 to 150 meV and aligned with theoretical calculations for rounded icosahedra AuNP dimers featuring two different types of gap (tip to face, face to face) and two different spacings (1 nm, 2 nm) (Fig. 10h). It should be noted that only 3% of the studied dimers displayed hybrid coupled modes, potentially due to the transition dipoles of emitters not being appropriately oriented or positioned on the flat surfaces of the AuNPs.

Note that this study also conducted electrodynamic simulations to emphasize the impact of nanoscale tips of polycrystalline Au nanoparticles on the likelihood of observing this type of coupling. To achieve more reproducible results, hybrid conditions such as particle crystallinity, size, and shape need further optimization. It remains to be determined whether similar systems have the potential to produce strong enough coupling with a single emitter, which could lead to various exciting quantum optical experiments.

## Strong coupling in nanoparticle-on-a-mirror

Nanoparticle-on-a-mirror (NpoM) is one of the robust cavities supporting gap plasmons, in which a small nanoparticle is placed atop a flat mirror surface. Plasmonic coupling in NPoM systems arises when the collective oscillations of electrons in a metallic nanoparticle resonantly couple with the electromagnetic field at the metal surface, subsequently influencing the scattering and absorption properties of the nanoparticle. This interaction can be modeled using image dipoles, which are virtual charges that emerge due to the reflection of the surface charges of nanoparticle in the metal mirror, thereby creating a system resembling a coupled plasmon dimer. For instance, in a nanosphere-on-mirror (NSoM) structure, where an isotropic metal nanosphere is positioned on a flat metal surface, a dipole oriented perpendicular to the surface couples in phase with its image dipole. This coupling enhances the electromagnetic field in the gap by nearly two orders of magnitude and tightly confines the fields to spatial volumes V < (6 nm)^3^, resulting in a high local density of optical states (LDOS) within the gap [89]. Closer proximity between the nanoparticle and the mirror surface can lead to stronger near-field interactions, similar to those in nanoparticle dimers. This configuration is versatile and relatively simple, as well as cost-effective to fabricate, as it does not necessitate complex fabrication processes or expensive materials. The effect of nanoparticle shape—such as sphere, rod, and cube—on the performance of an NPoM antenna has been both theoretically and experimentally investigated. Consequently, NPoM structures have been widely utilized in various applications, including biosensing, spectroscopy, and photovoltaics [90–94].

In this section, we explore strong coupling in NPoM systems examining interactions with molecular emitters and TMDC materials. Factors such as material composition, nanoparticle size and shape, and the refractive index of the spacer contribute to the variability in optimal gap thickness for Rabi splitting.

### Coupling with J-aggregates

**Fig. 11:**
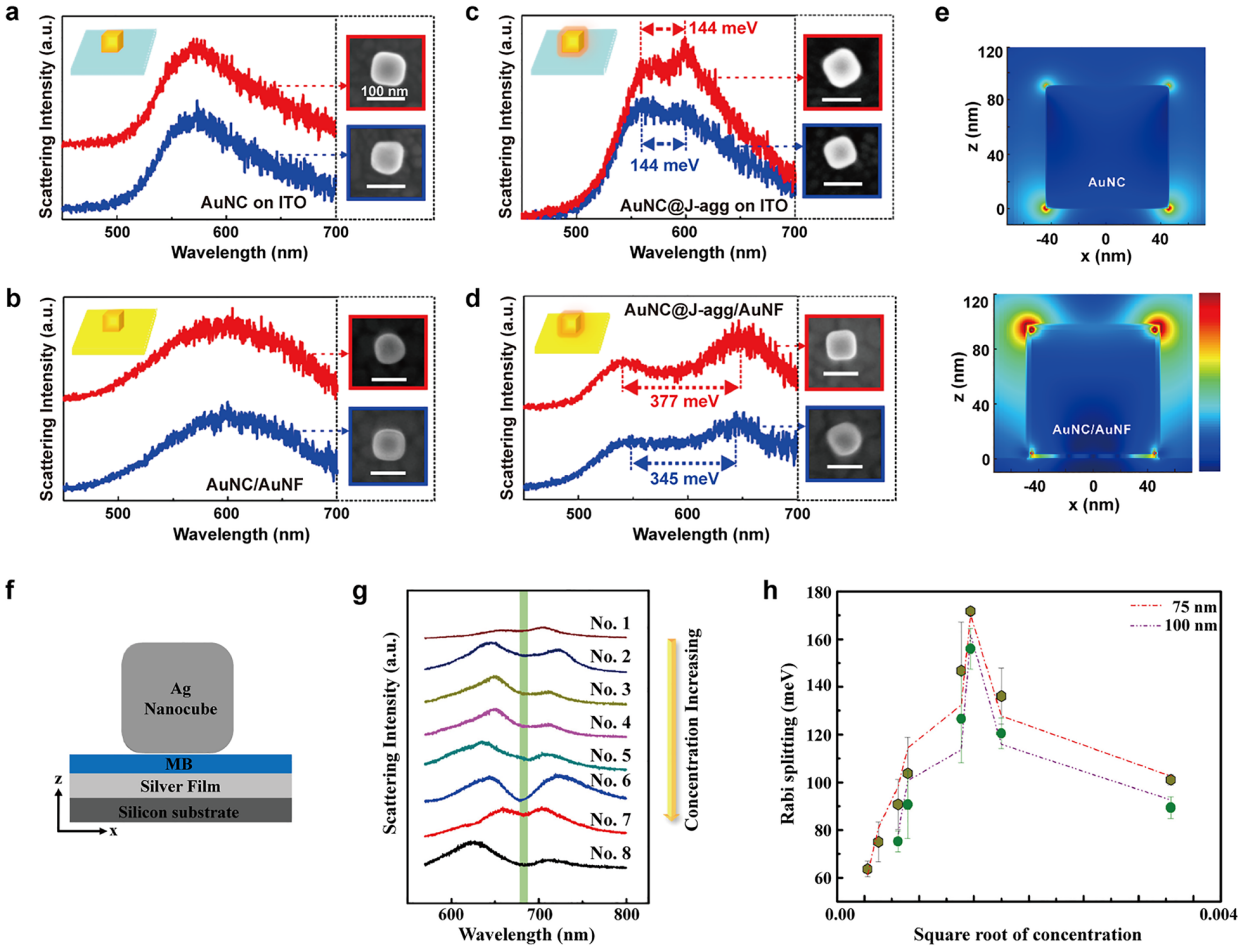
Strong coupling between nanoparticle-on-a-mirror and organic emitters. Scattering spectra of pure AuNCs on **a** ITO and **b** AuNF substrates. Scattering spectra of AuNC@J-aggregates hybrids on **c** ITO and **d** AuNF substrates. **e** Electric field distributions of the structures of (top) pure AuNC and (bottom) AuNC on AuNF. **f** Cross-sectional diagram of NPoM. **g** Scattering spectra of the NPoM in different concentrations of MB J-aggregates and 75 nm AgNC. **h** Rabi splitting as a function of the square root of MB concentration with different sizes of AgNC. Reproduced with permissions from [32, 95], respectively

The NPoM structure, consisting of Au film (AuNF) and AuNCs, was reported by Song et al. [32]. This example relates to a study that investigated the strong coupling between AuNCs and PIC J-aggregates, as mentioned in Sect. 2.1.1. The incorporation of NCs enables the creation of more intricate and complex structures, thereby enhancing the understanding of the strong coupling phenomenon. After the successful coating of AuNC with J-aggregates to confirm the presence of strong coupling, the resulting hybrids, AuNC@J-aggregates, were transferred onto the surface of an Au nanofilm. The scattering spectra obtained from pure AuNC on both the ITO substrate and the AuNF substrate consistently showed a single scattering peak (Fig. 11a, b). In contrast, the AuNC@J-aggregates hybrids on both ITO and AuNF substrates exhibited peak splitting, indicative of strong coupling (Fig. 11c, d). Notably, the Rabi splitting observed in the AuNC@J-aggregates/AuNF systems was approximately 2.5 times greater (measured between 345–377 meV) compared to the AuNC@J-aggregates hybrids on the ITO substrate, highlighting an enhanced plasmon-exciton coupling effect facilitated by the presence of the Au nanofilm. Simulations using the FDTD method demonstrated significant electric field enhancements at the two upper corners of the AuNC/AuNF system (Fig. 11e). This observation differs from the conventional understanding of maximum field enhancement occurring at the gap of nanostructures. Instead, it highlights the significant role played by the two upper corners in the AuNC/AuNF system for achieving substantial enhancements of coupling strength.

He et al. constructed the NPoM structure consisted of a Ag film and Ag nanocubes (AgNCs) (Q factor of ~ 13) (Fig. 11f) [95]. In the middle, a methylene blue (MB) solution was spin coated to form J-aggregates as a spacer layer then 75-nm-sized AgNCs were distributed on MB layers using polydimethylsiloxane to prevent ethanol in the nanocubes samples from damaging the dye molecules. The mode splitting was evident in DF measurements with a Rabi splitting of ~ 170 meV. The authors analyzed the impact of MB concentration and nanoparticle size on the coupling. All normalized spectra displayed two peaks and a dip at the exciton resonance of MB which occurs at 680 nm. The splitting appeared as a deeper dip with a nearly symmetrical line shape (Fig. 11g). When the MB concentration was below 1.5 × 10^−6^ mol/L, the thickness of MB changed from 4.2 to 9.0 nm as the MB concentration increased, and the coupling strength increased with increasing MB concentration (Fig. 11h). At higher MB concentrations, the magnitude of coupling strength decreased, and the thickness of the MB also decreased from 9.0 to 5.9 nm. The authors explained that this is due to the formation of a new type of dimer exciton at high MB concentrations, leading to a decrease in the number of MB monomers involved in strong coupling. The relationship between nanoparticle size and coupling strength was further studied with 100 nm AgNCs, and the results were consistent with those obtained with 75 nm nanocubes. However, it is still unclear that how higher MB concentration results in thinner J-aggregate layers and what impact the reduced number of excitons and thinner layers have on the changes in coupling strength in a quantitative manner.

### Coupling with dye molecules

**Fig. 12:**
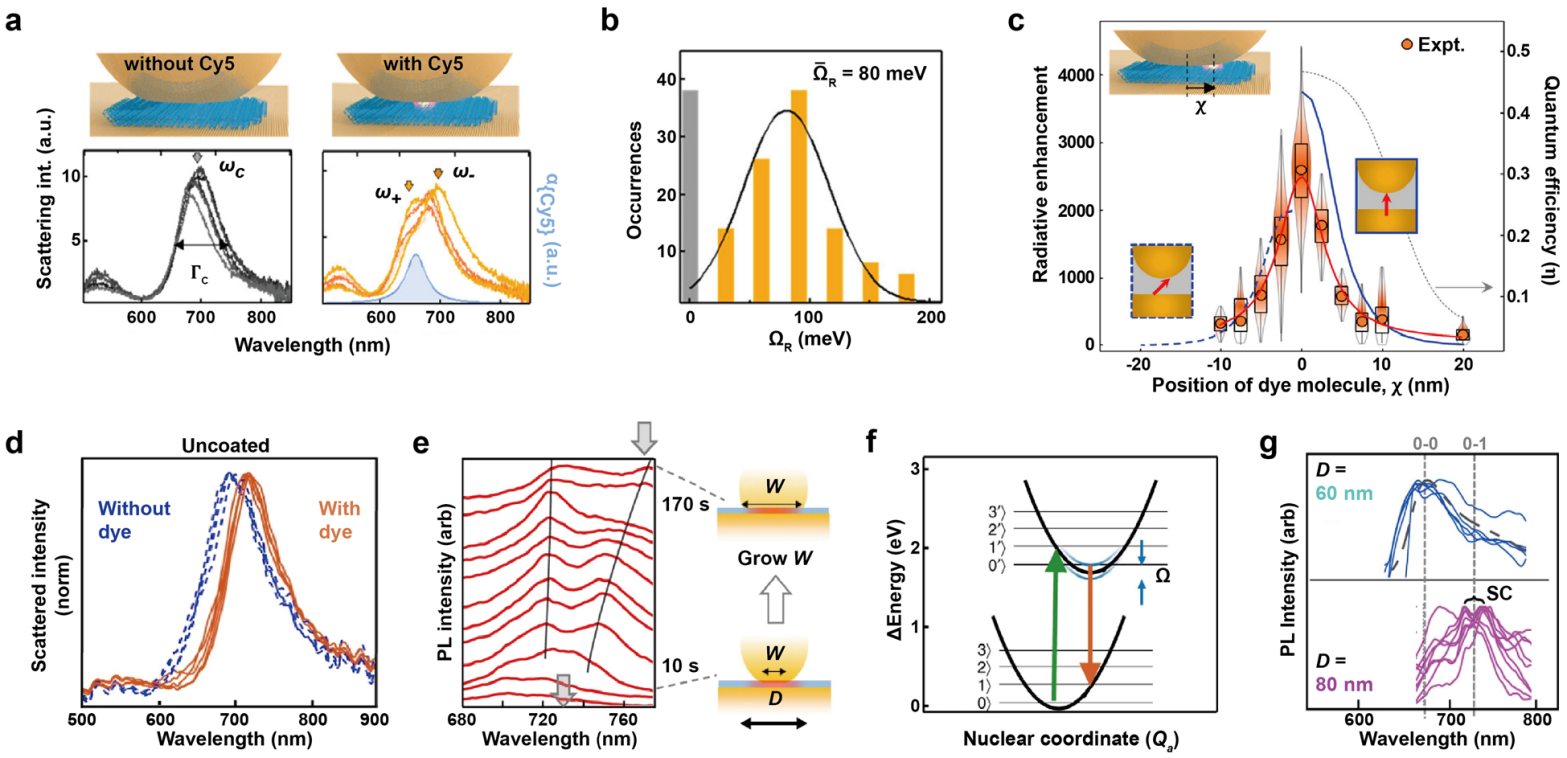
Strong coupling between nanoparticle-on-a-mirror and molecular emitters. **a** Schematics and scattering spectra without and with Cy5 molecule in DNAo. **b** Distribution of calculated Rabi splitting. **c** Experimental variation in emission enhancements with lateral displacement of single dye molecule. **d** Scattering spectra of ParlyeneC-uncoated NPoM without and with Atto647 molecule. **e** Time evolution of emission spectrum of Atto647 molecule in nanocavity structure and schematics. **f** Diagrams of the electronic and vibrational energy levels of the single molecule. **g** Scattering spectra of NPoM with different nanoparticle diameters. Reproduced with permissions from [6, 96], respectively

NPoM structure constructed with DNA origami can offer both strong light confinement and precise positioning of optically active single quantum emitters. Unlike DNA origami-based dimers having ~ 5 nm gap were coupled with randomly distributed J-aggregates in the Sect. 3.1.1, Chikkaraddy et al. demonstrated that a self-assembly method based on DNA origami structures can accurately place a single molecule in a sub-5 nm gap [96]. Without the Cy5, a characteristic infrared resonance peak of an empty cavity was identified at 702 ± 18 nm (Fig. 12a, left). By positioning the single molecules in the center of a nanocavity, they observed clearly resolved two peaks from > 200 NPoMs (Fig. 12a, right). The distribution of extracted coupling strengths for all NPoMs gives a mean Rabi splitting Ω = 80 meV (Fig. 12b). Interestingly, the authors found that the presence of a single Cy5 molecule not only disrupted the cavity scattering, but it also enhanced the optical emission from the Cy5 molecule due to the high LDOS in the gap. The fluorescence enhancement values were able to directly give a spatial profile of the LDOS with a resolution of ± 1.5 nm (Fig. 12c). The experimental data was also consistent with full 3D electromagnetic simulations for radiative enhancement with two different dipole orientations (90° and 45°, shown as solid and dashed lines, respectively), giving an actual dipole orientation of ~ 65°. Thus, this work not only provides the polariton formation from a hybridization between a single molecule and a single nanostructure, but also provides a simple and non-invasive way to measure and quantify confined optical modes at the nanoscale. It would be intriguing to examine the impact of increasing the number of molecules on the coupling strength with both isotropic and anisotropic particles since these two have different nanoscale hotspot distributions.

The same group further investigated the relationship between ultrafast pulses and a single molecule that was carefully assembled and connected to an ultralow volume plasmonic nanocavity at room temperature [6]. In this work, the authors created a resonant plasmonic mode by combining an 80-nm Au nanosphere with a underlying Au film. The gap between the two was filled with DNA origami that held a single Atto647 dye in place at the center of the gap. The entire sample was covered with a 30 nm layer of Parylene-C polymer to protect the Atto647 from the damaging effects of singlet oxygen. The gap was ~ 4.5-nm thick with a refractive index of ~ 2.1. The plasmonic mode had a volume of about 65 nm^3^ and the electromagnetic field was boosted by about 170 times. The presence of a single molecule did not result in two distinct peaks, but instead caused a 20 nm shift in the scattering resonance (Fig. 12d) due to the mismatch between the plasmonic scattering frequency (720 nm) and the absorption of the molecules (647 nm). In this near strong coupling regime, when the samples were irradiated with 120 fs, 80 MHz repetition rate pulses at 590 nm, Purcell enhancements > 1000 was observed with emission lifetime decreased from ~ 2.5 ns to < 0.3 ns due to high density of photonic states as seen in the earlier example [96]. When the pump power was increased, the emission intensity suddenly rises by four times after 20 s, and then doubles again after 50 s (Fig. 12e). Indeed, this continuous pumping caused the facet size of the nanoparticle to grow, resulting in DF scattering resonance shifts to 780 nm. This proved that the morphology change improves the molecular coupling which, in turn, resulted in an appearance of evolving two peaks in the PL with Ω ~ 30 meV. It is important to note that the strong coupling occurred with a vibration transition, which altered the branching ratio between 1′ → 0′ and 2′ → 0′ relaxations and therefore changed the emission spectrum (Fig. 12f).

When the particle size changed from 80 to 60 nm, the plasmon resonance aligned with the absorption frequency, causing peak splitting in DF scattering spectra. However, the emission spectrum now matched that in solution without any noticeable modifications from strong coupling (Fig. 12g). Although the full demonstration of this phenomena was limited by the risk of damaging the molecule and the light-assisted motion of Au atoms within the NPoM cavity, this study shows great potential for using advanced nano-assembly techniques to produce single-molecule quantum emitters that can operate at room temperature in normal conditions.

So far, strong coupling has been routinely accessed by spectral measurements but it is challenging to monitor coupling strength dynamically during the transition from strong to weak coupling regimes for multiple systems at the same time. Yuan et al. developed a far-field imaging technique that can directly observe the optical coupling dynamics in plasmon-exciton systems, allowing for the characterization of multiple nanocavity emissions from weak to strong coupling regimes [97]. In this work, Chlorophyll-a, a light-harvesting biomolecule, was used to study dynamic light-matter interactions in strongly coupled plasmonic nanocavities with a Au film and AgNCs. The dispersion curve's anticrossing behavior and large energy splitting, extracted from DF spectra, confirmed the strong-coupling regime with averaged coupling strength of ~ 125 meV. Then by analyzing red, green, and blue (RGB) values from DF images, the authors found out that the R/G ratio decreases as the coupling strength increases. In addition, the enhancement factor (EF) from fluorescent images decreased when the coupling strength exceeded 90 meV because the fluorescence enhancement by Purcell factor was not valid anymore when the light-matter interactions in the nanocavities became too strong. On the basis of these findings, dynamic imaging of the coupling strength was achievable using far-field images through observing the changes in the far-field images during photobleaching caused by UV illumination. These results indicated that the far-field imaging method can serve as a straightforward and efficient way to monitor delicate changes in coupling dynamics in plasmon-exciton systems. The proposed imaging-based, spectrometerless approach provides an alternative for future applications in quantum biosensing and imaging.

### Coupling with 2D transition metal dichalcogenides

**Fig. 13:**
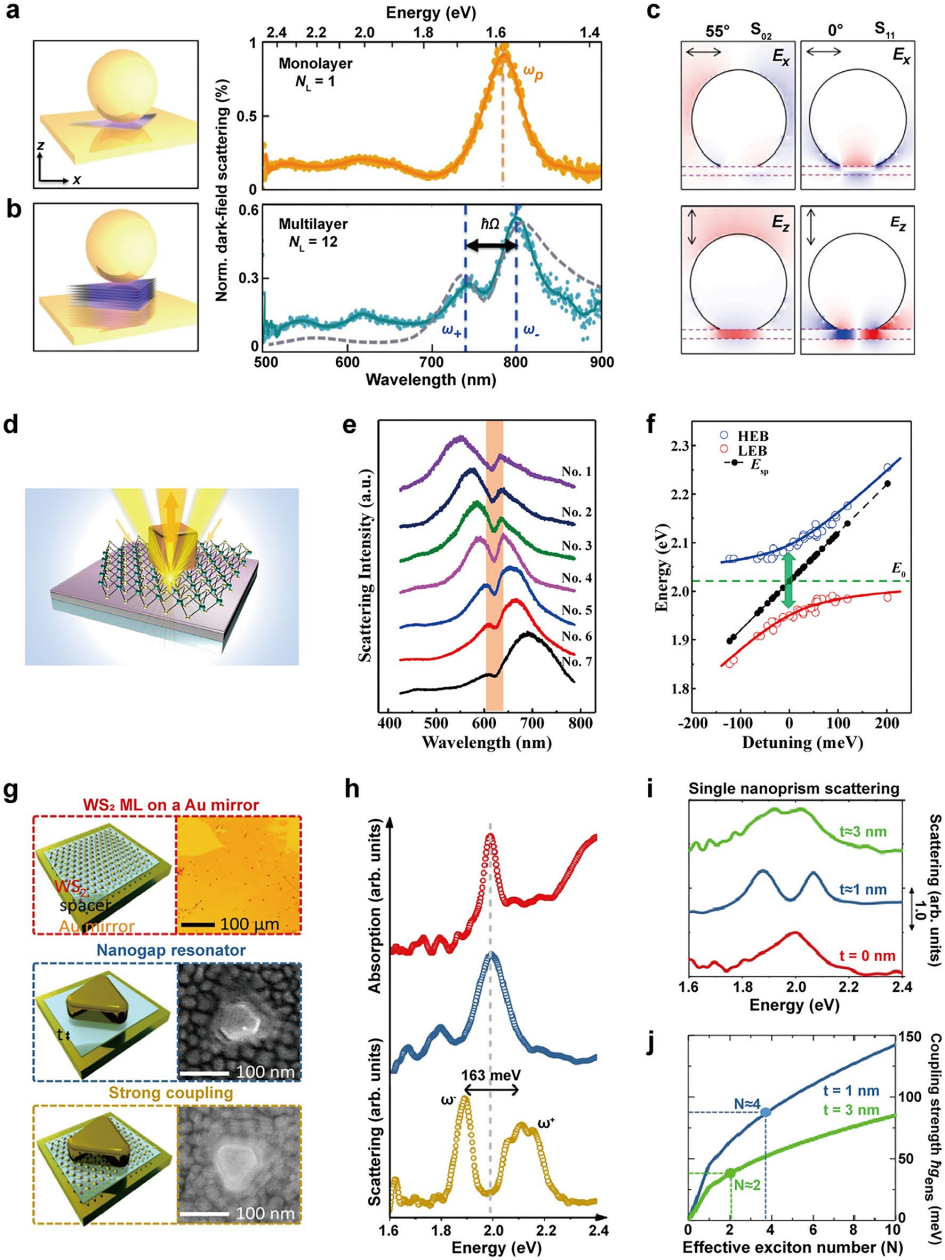
Strong coupling between nanoparticle-on-a-mirror and transition metal dichalcogenides. NPoM coupled to the **a** WSe_2_ monolayer and **b** multilayer and the corresponding scattering spectrum. **c** Simulated field distributions for different plasmon-coupled mode. **d** Schematic of measuring DF scattering signals of individual nanoparticles. **e** Scattering spectra of the hybrids with varying sizes of AgNC. **f** Dispersion of polaritons as a function of detuning. **g** Schematics with optical microscopy and SEM images of WS_2_ monolayer deposited on Au mirror, nanogap resonators, and strongly coupled system. **h** Absorption spectrum and scattering spectrum of each structure in **g**. **i** Scattering spectra of nanogap resonator with WS_2_. **j** Coupling strength as a function of exciton number. Reproduced with permissions from [98, 100, 103], respectively

In the first example, Kleemann et al. used the polycrystalline spherical AuNPs (diameter = 60–100 nm) to build a NPoM construct with Au film [98]. Spheres were selected since they supported blue-shifted resonances compared to cubes even with the high refractive index of TMDCs. Embedding a single WSe_2_ monolayers (*N*_*L*_ = 1) did not alter the plasmon resonance, exhibiting only a single peak beyond 700 nm (Fig. 13a). By incorporating thicker WSe_2_ flakes (thickness 10 nm with *N*_*L*_ = 12 layers), a splitting was seen with a Rabi splitting of ~ 140 meV (Fig. 13b). Although TMDCs are highly anisotropic supporting strong in-plane excitons, the E_x_ field strengths aligned to the dominant in-plane exciton dipole orientation were insufficient to retrieve strong coupling (Fig. 13c). In multilayers, exciton dipole strength along z became of order 25% of the x-dipole for multilayers, thus offering a change for coupling with strongly confined E_z_ gap plasmon modes.

Further, strong coupling of single TMDC layers in NPoM were also demonstrated. Hou et al. proposed a nanoantenna created using AgNC positioned on top of Au film and separated by a dielectric MoS_2_ layer [99]. When the spacer layer was thick (> 10 nm), a 1570-fold enhancement in PL was observed at the weak coupling regime in hybrid nanocavities. By decreasing the thickness of the spacer layer, transition from weak coupling to strong coupling between excitons and plasmons occurred with Rabi splitting up to 190 meV at ~ 5-nm spacer thickness. The authors conducted numerical calculations to examine the relationship between the strength of coupling, the density of states in a specific area, and the thickness of the spacer. As the spacer in nanocube-on-mirror (NCoM) became gradually thinner, the size of the mode volume decreased significantly due to the strong confinement of plasmon modes. This resulted in a significant increase in the extracted g value, and strong coupling was achieved when the spacer was less than approximately 6-nm thick. However, when the spacer was thinner than 5 nm, the dominant form of decay was nonradiative, due to metal absorption. Therefore, NCoM with a 5-nm spacer was the optimal conditions for strong coupling in this system.

Han et al. explored the coupling between WS_2_ and the NPoM nanocavity made up of a AgNC and a Ag film with a few-nanometer-thick Al_2_O_3_ spacer (Fig. 13d) [100]. Like MoS_2_, WS_2_ monolayers are direct bandgap materials having different bandgap energies; MoS_2_ has a bandgap energy of around 1.8 eV [101] and WS_2_ has a slightly larger bandgap energy of around 2 eV [102]. WS_2_ is known to have a higher oscillator strength compared to MoS_2_. The plasmonic resonance of the nanocavity could be adjusted by controlling the size of the AgNCs from 65 to 95 nm, making it possible to precisely match the exciton energy of the WS_2_ monolayer (Fig. 13e). The mode splitting was clearly visible in the DF scattering spectrum of the single hybrid nanocavity with a Rabi splitting of ~ 145 meV (Fig. 13f). Since the coupling strength can be described as $\mathrm{g}= {\mu }_{m}\sqrt{\frac{4\pi \hslash Nc}{\mathrm{\lambda \varepsilon }{\varepsilon }_{0}V}}$ where μ_m_ = 56D is the exciton transition dipole moment of WS_2_ monolayers; 27 N, λ, ε, and V are the number and wavelength of exciton, dielectric function, and mode volume, respectively, and effective mode volume in the plasmonic nanocavity can be calculated as V = 0.001(λ/n)^3^, the authors estimated about the effective exciton number N = 130.

Qin et al. furthermore explicitly controlled the exciton strength coupled to NPoM gap plasmons by adjusting the spacing between the 2D material monolayers and the metal film [103]. Here the nanoresonator was constructed by placing a Au nanoprism on top of the Au film with a WS_2_ monolayer (absorption maximum at 1.99 eV) as a dielectric spacer (Fig. 13g). While WS_2_ monolayer on a Au mirror and empty nanogap resonator both exhibited single peaks in their absorption and scattering spectra respectively, a WS_2_ monolayer embedded in a nanogap resonator with a spacer thickness t ≈ 1 nm exhibited a splitting feature of strong coupling between plasmons and excitons with Rabi splitting of ~ 163 meV (Fig. 13h). Slight modification in the spacer thickness to 0 nm with the same WS_2_ layer made a system supporting only a single peak (Fig. 13i) because the direct contact of WS_2_ monolayers with a metal film significantly suppressed the formation of the Wannier-type excitons due to the enhanced charge transfer from TMDCs to metals. When the spacer thickness increased to ~ 3 nm, the system displayed a broadened spectrum with a very shallow mode splitting. Note that here the electromagnetic environment was controlled by changing the spacer thickness while it could be done by controlling the nanoparticle size as in the earlier example [100].

The authors also estimated the effective exciton number N coupled to the cavity based on numerical calculations of V and F and by using the equation, ${\mathrm{g}}_{ens}=\sqrt{\frac{\omega }{\hslash {\varepsilon }_{0}\mathrm{Re}[\frac{d(\omega \varepsilon )}{d\omega }]V}NF}{d}_{0}$, where N stands for the effective exciton numbers contributing to the coupling process, d_0_ = 1.86 × 10^−28^ C m refers to the electric dipole moment of exciton, V is the mode volume, and the normalized factor F reveals the integrated plasmon-exciton coupling efficiency toward different orientations at different positions (See Supplemental Material at [103]). When the spacer thickness was ~ 1 nm, the average coupling strength of 82 meV was equivalent to an exciton number of 4 (Fig. 13j, blue curve). When the monolayer-Metal distance was increased to 3 nm, the average coupling strength decreased to 38 meV and the estimated exciton number becomes 2 (Fig. 13j, green curve), which was close to exciton unity. Hence, simply changing the electromagnetic environment in nearby semiconductor monolayers enabled the modification of exciton strength at the same WS_2_ monolayer.

However, although the authors mentioned that these calculations might not provide an exact determination of N due to missing resonator information, the number of excitons provided here is 2 orders of magnitude lower than previous systems, which is a large mismatch considering the same WS_2_ monolayer and similar cavity architecture [100]. Thus, more detailed analysis is required to compare these two systems by their types of metals, size and shape of the particles, mode volumes, electromagnetic field intensity, etc.

In summary, in NPoM systems, the optimum gap thickness yielding the large splitting varied from 1 to 9 nm. The differences may originate because the cavity resonances are highly dependent on the material, size and shape of the particles, and the refractive index of the spacer. The number and the orientation of excitons also affect the interaction between plasmons and excitons. Compared to the nanoparticle dimers, NPoM structures show similar level of Rabi splitting in the case of J-aggregates and TMDCs as expected by the similarity between two cavity systems. However, in the case of non-aggregated dye molecules, NPoM demonstrated the strong coupling with even a single emitter whereas nanoparticle dimers have been coupled at least a few molecules. Thus, it would be interesting to directly compare the two systems by using exactly same type of particles and excitonic materials.

## Strong coupling in other types of nanoparticle-based resonators

### Tip-based strong coupling

**Fig. 14:**
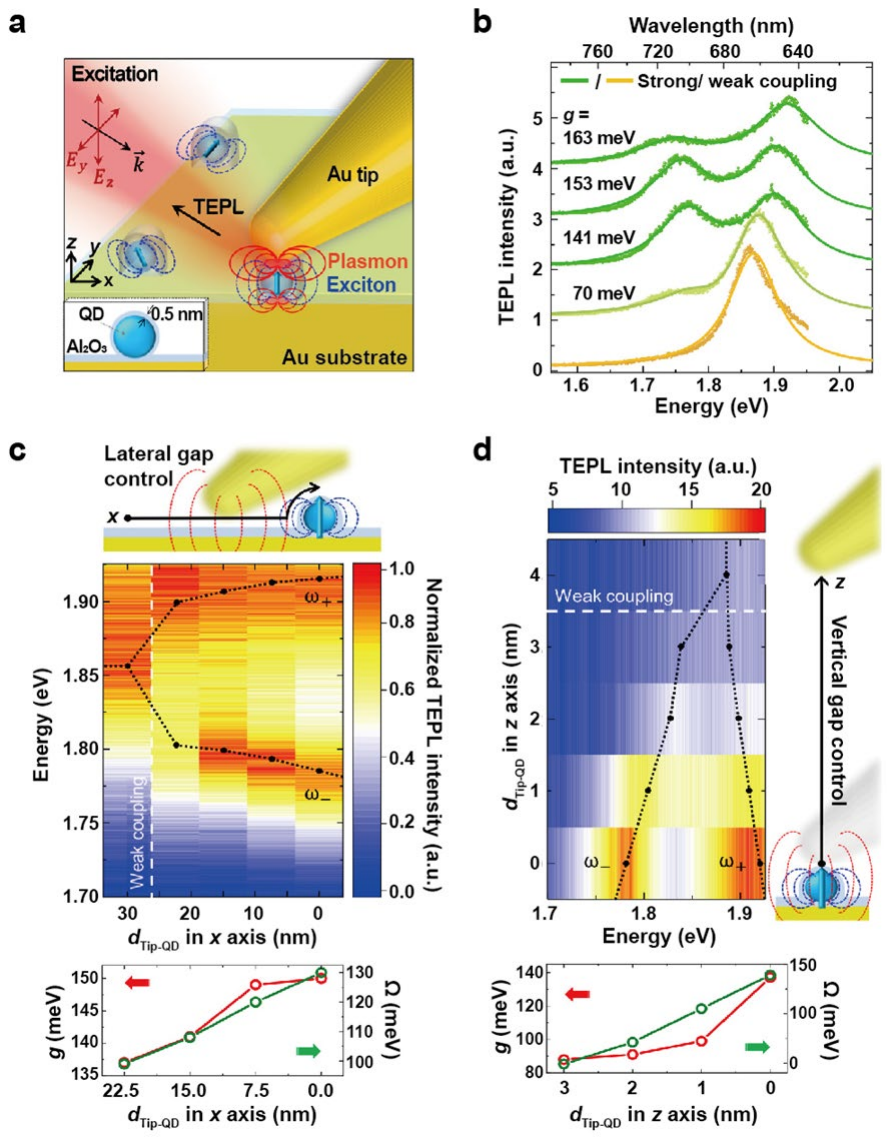
Strong coupling in tip-based nanoparticles. **a** The strongly confined ∣E_z_∣ fields in a single CdSe/ZnS QD with Al_2_O_3_ layer and Au tip. **b** TEPL spectra of different single QDs with varying coupling constant and Rabi frequency. TEPL spectra were obtained with varying **c** lateral tip-QD distances and **d** vertical distances. Reproduced with permissions from [104]

Recently, tip-enhanced light-matter interactions uncover the strong coupling between quantum emitters and nanocavities. As a sharp scanning metal tip is positioned close to the metal surface and acts as a local electromagnetic field enhancer in Tip-enhanced Raman spectroscopy (TERS), a nanocavity formed between the tip and the substrate couples with a single emitter and creates hybrid quantum states in Tip-enhanced strong coupling (TESC). Park et al. drop-casted single isolated CdSe/ZnS QDs on a template-stripped Au film and covered the sample with a thin Al_2_O_3_ layer to prevent photo-oxidation of QDs (Fig. 14a) [104]. The resulting plasmonic cavity supported strong light confinement in out-of-plane direction couples to single QDs, exhibiting two peaks in PL spectra with mode splitting from 70 to 163 meV (Fig. 14b). In addition, the authors took advantage of TESC to precisely control the position of the tip provides a means of tuning the coupling strength and mode volume of the nanocavity. Figure 14c shows how the strong coupling regime is entered with a rise in Rabi splitting up to g of ~ 140 meV, as the tip-QD distances are varied from 30 to 0 nm. On the other hand, controlling the vertical distance between the tip and the sample with sub-nanometer precision (Fig. 14d) demonstrated the high level of spatial confinement, as the gap width decreased from 4 to 0 nm. The shorter length scale when compared to the lateral distance between the tip and QD was because of the sharp decrease in coupling strength. Thus, here although emitters were in static positions, TESC demonstrated new possibilities for controlling quantum dynamics through nanoscale tip positioning. This approach can be generalized to any optical modality and extended further through different techniques, ranging from larger emitters to nanoplasmonic tip engineering. It also provides a new means of tuning quantum-optical interfaces, enabling active and dynamic control of photochemical pathways at the single-molecule level.

### Coupling in chiral materials

Previous studies on the strong interaction between excitonic materials and plasmonic nanostructures have mainly focused on achiral materials. However, the study of chiral light-matter interaction advances the understanding of chiral quantum optics, providing the potential to facilitate the development of chiroptical devices in the fields of nanophotonics and information communication.

**Fig. 15:**
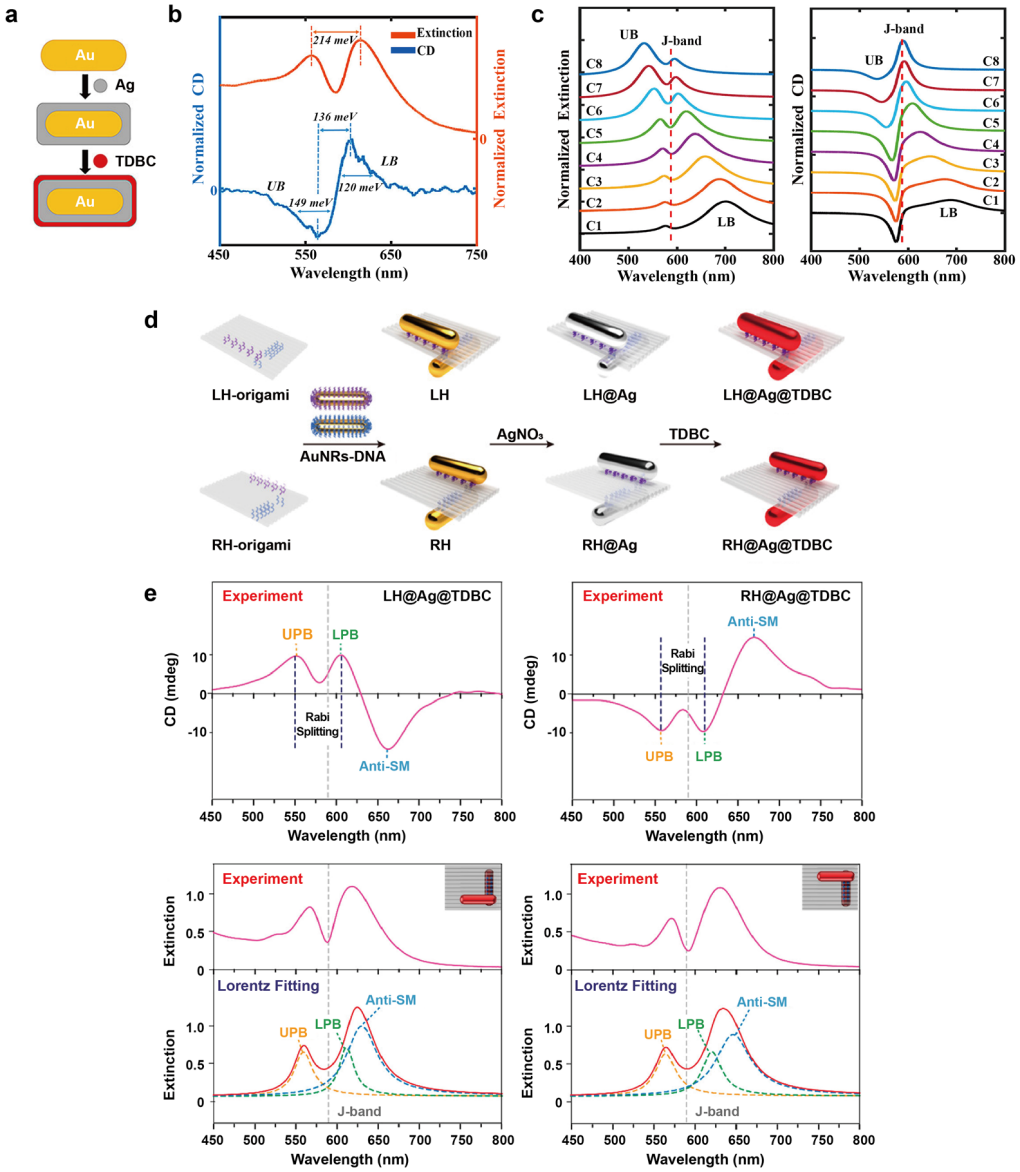
Strong coupling using chiral materials. **a** Au@Ag nanocuboids coupled to the chiral J-aggregates. **b** Extinction and CD spectra of the hybrids system. **c** Calculated (left) extinction and (right) CD spectra of the hybrid system. **d** Diagram of the fabrication of plasmonic-excitonic chiral hybrids with nanorod dimer. **e** Experimental (top) CD spectra and (bottom) extinction spectra and the corresponding Lorentz fitting of (left) LH@Ag@TDBC and (right) RH@Ag@TDBC structures. Reproduced with permissions from [105, 106], respectively

Chirality properties of simple polaritonic systems can be explored by circular dichroism (CD) spectroscopy [105]. When chiral molecules were attached to an achiral Au@Ag nanocuboids through electrostatic interaction (Fig. 15a), the coupling between plasmons and excitons can significantly affect the CD responses of the hybrid system. TDBC molecules were used as chiral molecules. Although the monomer molecule of TDBC is achiral, the CD spectrum of TDBC J-aggregates occurring around the J-band (~ 586 nm) exhibit optical activity, which is expected to be due to the chiral arrangement of monomers.

When the Ag shell thickness of Au@Ag nanocuboids was adjusted to match the plasmon resonance with the absorption band of J-aggregates, the extinction spectrum exhibited two spectral branches, the upper polariton and the lower polariton. Focusing on the CD spectrum of the hybrid systems, the authors confirmed that a bisignate signal containing two opposite sign peaks on both sides of the J-band occurs in the CD spectrum. Both the extinction and CD spectra of the hybrid nanosystems exhibited mode splitting and anticrossing behavior, indicating strong coupling. The CD and extinction measurements revealed a significant mode splitting of up to 136 meV/214 meV at zero detuning (Fig. 15b). Although, the mode splitting observed in the CD response was smaller compared to the extinction response, the hybrid modes can be more easily distinguished in the CD response due to the smaller line width and bisignate line shape. The calculations of extinction and CD responses agree with the experimental observations of mode splitting and anticrossing behavior (Fig. 15c).

Zhu et al. used AuNRs to construct dimeric structures with DNA origami [106]. Unlike dimers made from spherical particles, the asymmetrical shape of AuNRs affects the symmetry of the dimers, allows to bring chirality in strong coupling. By assembling the nanorods either clockwise or anticlockwise, the dimers were divided into left-handed or right-handed plasmonic cavities, respectively (Fig. 15d), that support two adjacent resonance modes, symmetric mode (SM) and antisymmetric mode (anti-SM). It is important to note that these plasmon modes show strong but different circular dichroism with large nonlinear optical responses. Once coated with TDBC J-aggregates, the J-band was coupled to the SM mode, resulting in Rabi splitting and anticrossing behavior in the CD spectra, indicating chiroptical hybridization. The left-handed and right-handed plexcitonic nanosystems exhibited Rabi splitting of 205 meV and 199 meV, respectively, and the CD signals had opposite signs due to the different chiral handedness (Fig. 15e, top). In extinction spectra, it was difficult to distinguish Rabi splitting because of the overlap between lower polariton bands and anti-SM modes (Fig. 15e, bottom). These results demonstrated that CD spectroscopy can be more helpful in characterizing chiral hybrids with multiple adjacent plasmon resonance modes.

The exploration of optical chirality in strong coupling, achieved through the chiral arrangement of organic dyes or plasmonic nanoparticles, adds interest to this field of study. Active research is currently underway to develop chiral nanostructures by precisely arranging chiral nanoparticles in specific positions and orientations using templates or scaffolds [107, 108]. The advancement of synthesis techniques for chiral nanostructures will hold significant promise in driving further progress in the field of chirality in strong coupling and exploring diverse applications across sensing, catalysis, and the development of novel drugs.

## Challenges and perspectives

The study of strong coupling in metal nanoparticles has demonstrated significant potential in advancing the understanding and application of plasmon-exciton interactions across various types of nanostructures and excitonic materials. As research in this area continues to progress, there are several key perspectives for further exploration and investigation.

One of the advantages of metal nanoparticles is their readily tunable plasmonic resonances by changing the size, shape, and material of the nanoparticles. However, synthesized particles have certain level of size and shape distributions. Thus, the preparation of metal nanoparticles with well-controlled sizes and shapes is crucial for achieving strong coupling in a reproducible and reliable manner. Precise positioning of emitters at specific locally enhanced areas and probing the exact locations attached on the nanoparticles are also critical to control over the coupling strength and to make them more versatile for different applications.

The strong coupling of individual emitters to plasmonic modes offers exciting opportunities for quantum optical experiments and devices. Both dimer configurations and NPoM systems provide conducive environments that facilitate strong coupling even with individual emitters. Further research into it could open up the coupling of chemistry to quantum information processing and communication, and tailoring of the excited state manifold for novel control of chemical reactions. While NPoM systems are distinguished by their ability to create more homogenous and robust local fields, their scalability and inherent constraints pertaining to the limited excitation angle must be thoughtfully considered. The selection of materials further introduces an additional dimension of consideration. For instance, systems based on Au cannot sustain plasmon modes above approximately 520 nm. Furthermore, plasmonically coupled structures tend to generate red-shifted plasmon modes. Therefore, a careful evaluation of the plasmonically active wavelength range is required, contingent upon the specific requirements of the nanostructures and emitters under study.

Achieving ultrastrong coupling in metal nanoparticles is challenging. Ultrastrong coupling represents a regime where the interaction strength between plasmons and excitons becomes comparable to the resonance frequencies of the interacting particles themselves. This regime is associated with unique phenomena and holds promise for various applications in quantum information processing, sensing, and photonics. However, metal nanoparticles inherently suffer from ohmic losses due to the absorption of energy by free electrons within the metal. These losses result in reduced quality factors and limit the coupling strength that can be achieved. To overcome this challenge and reach the ultrastrong coupling regime, tailoring nanoparticle geometry and composition or using low-loss plasmonic materials could be considered.

Finally, exciton-polaritons have found applications in various fields such as gain materials for lasers, control of chemical reactions, and polariton-mediated energy transfer, particularly in the context of strongly coupled microcavities [109–111]. However, the applications involving colloidal plasmonic nanoparticles remain relatively understudied. Further exploration and investigation on nonlinear optical properties or Bose–Einstein condensation will be interesting to investigate in these colloidal nanoparticle polaritonic systems. When nanoparticle-based polaritons become available for enhancing or altering how molecules behave in chemical reactions, one may open a field of polaritonic nanoreactor, revealing new insights into the fundamental chemistry from light-matter interactions at the nanoscale.

## Data Availability

Not applicable.
